# CCL24 recruits CCR3^+^ TAMs to promote immunosuppression via YAP1 activation and serves as a therapeutic target for Gracillin in colorectal cancer

**DOI:** 10.7150/ijbs.127341

**Published:** 2026-01-30

**Authors:** Shiyu Huang, Wanqiong Lin, Xu Ding, Jinjun Shi, Fei Liu, Guoqing Wang, Chanchan Gao, Xiangyu Su

**Affiliations:** 1 School of Medicine, Southeast University, Nanjing, China.; 2 Department of Oncology, Zhongda Hospital, School of Medicine, Southeast University, Nanjing, China.; 3 Department of Ultrasound, Zhongda Hospital, Medical School, Southeast University, Nanjing, China.; 4 Department of Medical Oncology, Luhe People's Hospital of Nanjing, Nanjing, Jiangsu, China.; 5 Department of Pathology, Zhongda Hospital, School of Medicine, Southeast University, Nanjing, China.

**Keywords:** CCL24, CCR3, Colorectal cancer, TAMs, Gracillin, Immune evasion

## Abstract

**Background:** CC chemokines orchestrate intercellular communication and modulate tumor microenvironment. This study investigates the role of C-C motif chemokine ligand 24 (CCL24) in immune regulation in colorectal cancer (CRC).

**Methods:** CCL24 expression and its clinical relevance in CRC were analyzed via bioinformatics and tissue microarrays. Genetic knockout of CCL24, or antibody-mediated inhibition of CCL24 was performed in AOM/DSS-induced mouse CRC models. CCL24 knockout (CCL24^ko^) CRC cells were co-cultured with macrophages or CD8^+^ T cells. Mouse MC38 CRC cells with CCL24^ko^ were implanted into C57BL/6 mice to generate subcutaneous or metastasis models. Molecular docking was conducted to identify potential pharmacological inhibitors of CCL24.

**Results:** CCL24 is abundantly expressed in CRC tissues and linked to T cell dysfunction and unfavorable patient survival. Inhibition or knockout of CCL24 suppressed AOM/DSS-induced colorectal tumorigenesis in mice, reduced the population of tumor-associated macrophages (TAMs), and increased CD8^+^ T cell numbers. While the morphology of CCL24^ko^ cells showed minimal changes* in vitro*, their tumorigenic ability was reduced in immunocompetent but not in immunodeficient mice. CCL24 did not directly alter CD8^+^ T cell populations; instead, CCL24^+^ tumor cells recruited CCR3^+^ TAMs, which promote immunosuppression by promoting nuclear translocation of YAP1, a key transcription factor of the Hippo pathway. Gracillin, a natural compound, was identified as a CCL24 inhibitor and synergized with 5-fluorouracil and programmed cell death 1 monoclonal antibody therapies in allograft-bearing mice.

**Conclusion:** CCL24 facilitates recruitment of CCR3^+^ TAMs, enhancing the immunosuppressive TME in CRC. Targeting CCL24 with agents like gracillin represents a promising therapeutic strategy.

## Introduction

Colorectal cancer (CRC) ranks among the most prevalent cancers globally, contributing to roughly 10% of all cancer cases and deaths worldwide 1. In 2022, global statistics reported approximately 1.93 million new CRC cases, with over 900,000 fatalities 2. In China alone, nearly 600,000 cases and more than 40,000 deaths were recorded 3. Alarmingly, projections suggest that both the incidence and mortality rates of CRC will continue to climb 4. While advancements in treatments such as surgery, chemotherapy, radiotherapy, targeted therapies, and immunotherapy have significantly extended patient survival 5-8, the prognosis for CRC, particularly in cases of metastatic disease, remains suboptimal 9, 10. Survival rates vary starkly: patients with localized disease have a 5-year survival rate of about 90%, those with regional lymph node or nearby metastasis have a 70.4% rate, and those with distant metastasis face a mere 12.5% survival rate 11. This poor outcome is largely due to complex, adaptive therapy resistance mechanisms that limit the sustained efficacy of current treatments 12.

In their 2011 update, Hanahan and Weinberg expanded the hallmarks of cancer to include two key features of tumor development: rewired energy metabolism and immune evasion 13. This is particularly pertinent to CRC, often described as an "immune-cold" malignancy due to limited immune cell infiltration [14, 15]and poor responsiveness to immunotherapies 16, such as immune checkpoint inhibitors targeting programmed cell death protein 1 (PD-1) or its ligand programmed death-ligand 1 (PD-L1) 8. Within the tumor microenvironment (TME), tumor-associated macrophages (TAMs) are a dominant immune cell type in solid tumors, including CRC 17. Traditionally, TAMs are divided into pro-inflammatory (M1) and anti-inflammatory (M2) subtypes, but this binary model oversimplifies their complex and adaptable behavior within the TME 18. The M2-like subtype, identified by markers like CD206 (mannose receptor C-type 1, MRC1) or arginase 1 (Arg1), is typically dominant in the TME 19, 20. In contrast, tumor-infiltrating lymphocytes, especially CD8^+^ T cells, are associated with better prognosis across various solid tumors 21. These cells combat cancer by releasing cytotoxic molecules such as granzymes, perforin, and pro-inflammatory cytokines 22, but their effectiveness is often curtailed by immunosuppressive signals within the TME 23. Understanding the molecular drivers of this immunosuppression and developing novel treatment strategies are critical priorities for researchers and clinicians.

CC chemokines, a group of 27 chemotactic cytokines, play an essential role in intercellular signaling within the TME 24. These small secreted proteins primarily regulate leukocyte movement and immune cell activity 25. Among them, C-C motif chemokine ligand 24 (CCL24), also known as eotaxin-2, signals through the C-C motif chemokine receptor 3 (CCR3) and is instrumental in recruiting immune cells such as eosinophils, basophils, TAMs, and Type-2 helper T (Th2) cells 26. Compared to other chemokines, research on eotaxins, including CCL24, in cancer is sparse, with few studies exploring targeted therapies against them 24. Notably, CCL24 has been identified as a key chemokine linked to both primary and metastatic colorectal tumors 27. However, its specific role in CRC remains poorly understood. This study seeks to investigate CCL24's role in tumor progression, with a focus on its influence on TME modulation in the context of CRC. Additionally, Gracillin a natural steroidal saponin component abundant in several Dioscorea species 28, was identified as a molecule showing high affinity with CCL24 through molecular docking analysis. Saponins, including Gracillin, are known for their antimicrobial, antioxidant, anti-inflammatory, and anti-tumor properties 29-32. The influence of Gracillin in CCL24 activity, and its treatment potential for CRC management, will be investigated as well.

## Materials and Methods

### Cells and culture

Human CRC cell lines HCT116, Lovo, SW480, and mouse CRC cells MC38 were purchased from ViCell Biotechnologies, Shanghai, China, and normal human colon epithelial cells FHC were obtained from ScienCell Research Laboratories (CA, USA). The cells were cultured in high-glucose DMEM (for HCT116, SW480, MC38) or RPMI 1640 medium (for Lovo, FHC) supplemented with 10% fetal bovine serum (FBS, Gibco) and 1% antibiotics (Gibco). Cells were maintained at 37 ºC in a 5% CO_2_ incubator. For passaging, cells were digested with 0.25% trypsin (Gibco) at a splitting ratio not exceeding 1:5. All cells were tested for mycoplasma contamination before use, and the medium was refreshed every 2-3 days.

### THP-1 induction and co-culture experiments

The human monocytic leukemia cell line THP-1 (ViCell) was cultured in complete RPMI-1640 medium and induced to differentiate into macrophage-like phenotype by stimulation with 100 ng/mL phorbol-12-myristate-13-acetate (PMA, Sigma-Aldrich, Merck KGaA) for 48 h. After induction, cells were gently washed twice with PBS and maintained in fresh complete medium without PMA for 24 h to allow recovery. Subsequently, HCT116 cells were co-cultured with THP-1-derived macrophages in a Transwell system (0.4 μm pore size) for 48 h. Alternatively, mouse CCL24 recombinant protein (mCCL24, Peprotech, 100 ng/mL) was directly supplemented in the co-culture system to evaluate its functional role.

### CCL24 knockout cell construction

CRISPR/Cas9 technology was employed to knock out the CCL24 gene in HCT116 and MC38 cell lines. Specifically, sgRNA sequences targeting mouse or human CCL24 genes were designed using the CRISPR Design Tool and ligated into the lentiCRISPR v2 vector (Addgene #52961). Lentiviral particles were generated by packaging in 293T cells. Target cells were infected and selected in medium containing 2 μg/mL puromycin for 7 days to obtain stable knockout (CCL24^ko^) clones.

### *In vitro* cell proliferation and invasion assays

Cell proliferation was determined using a colony formation assay. Wild-type (WT) or CCL24^ko^ HCT116 or MC38 cells were seeded at 500 cells per well in 6-well plates and cultured for 10-14 days. Visible colonies were fixed with 4% paraformaldehyde for crystal violet staining. Colonies with a diameter > 1 mm were photographed and counted. Cell invasion was evaluated using Transwell chambers (Corning, 8.0 μm) pre-coated with Matrigel (Corning). Cells were suspended in serum-free medium and seeded in the upper chambers, with serum-containing complete medium loaded to the lower chambers as a chemoattractant. After 24 h of incubation, cells were fixed and stained. The number of cells in the upper chamber was counted to assess invasion capacity.

### Macrophage chemotaxis assays

Macrophage migration was evaluated using Transwell inserts (Corning, 8.0 μm pore size) without Matrigel coating. Macrophages (RAW264.7 1 × 10^5^ cells) were suspended in serum-free medium and seeded into the upper chamber. The lower chamber was filled with 600 μL of serum-free medium containing either: (1) recombinant mouse or human CCL24 protein (100 ng/mL, PeproTech); (2) conditioned medium (CM) collected from WT or CCL24^ko^ CRC cells (MC38 or HCT116) after 24 h of culture; or (3) medium supplemented with specific inhibitors. To validate the specificity of the CCL24-CCR3 interaction, macrophages were pre-treated with the CCR3 antagonist SB 328437 (10 μM, MedChemExpress) for 1 h prior to seeding, or the lower chamber media was pre-incubated with anti-CCL24 neutralizing antibody (2 μg/mL) for 30 min. The plates were incubated at 37ººC in 5% CO- for 24 h. Non-migrated cells on the upper surface were removed, and migrated cells on the lower surface were fixed with 4% paraformaldehyde and stained with 0.1% crystal violet. Migrated cells were imaged and counted in five random fields per well using a microscope.

### Cytokine detection

To evaluate the immunomodulatory status of tumor or immune cells under different treatment conditions, CCL24 levels of in the culture supernatant of cancer cells, arginase 1 (Arg1) levels in the culture supernatant of TAMs, and serum levels of CCL24, tumor necrosis factor alpha (TNFA), interferon gamma (IFNG), granzyme B (GZMB), PFN, and interleukin 2 (IL2) in mouse models (see details below) were determined using the Enzyme-linked immunosorbent assay (ELISA) kits (Elabscience Biotechnology or BioLegend). All procedures were conducted strictly adhering to the manufacturer's instructions. Nitric oxide (NO) levels in the supernatant of TAMs were determined using a Griess reagent kit (Beyotime) by colorimetric measurement of nitrite concentration to reflect NO release. Reactive oxygen species (ROS) levels were measured using DCFH-DA probe staining, followed by detection with a multifunctional microplate reader.

### T cell function and proliferation detection

The use of animals was approved by the Animal Ethics Committee of our Institute. Thymus from C57BL/6J mice (6 weeks old, Anburui Biotechnologies, Fujian, China) was mechanically dissociated to prepare a single-cell suspension, and CD8^+^ T cells were purified by positive selection using CD8a microbeads (Miltenyi Biotec). The sorted CD8^+^ T cells were activated with CD3/CD28 agonist antibodies (2 μg/mL, BioLegend) for 48 h. Cell function was assessed by intracellular cytokine staining and flow cytometry to evaluate TNFA and GZMB expression. Proliferation was measured by labeling T cells with carboxyfluorescein diacetate succinimidyl ester (CFSE) dye (Thermo Fisher Scientific) and co-culturing with MC38 cells or TAMs for 72 h. Data were collected using a BD LSRFortessa flow cytometer and analyzed with FlowJo software. Strict gating strategies were applied for all analyses to ensure data accuracy. First, cell debris and dead cells were excluded based on Forward Scatter (FSC) and Side Scatter (SSC) characteristics. Doublets were then excluded using FSC-Height versus FSC-Area plots. Live immune cells were identified as CD45^+^. For T cell analysis, cells were sequentially gated on CD3^+^ and then subdivided into CD4^+^ or CD8+ populations. For macrophage analysis, cells were gated on CD11b^+^ and F4/80^+^, and functional phenotypes were further identified using specific markers including CCR3, Arg1, and Mrc1.

### T cell cytotoxicity analysis

To evaluate the cytotoxicity of CD8⁺ T cells against CRC cells, activated CD8⁺ T cells were co-cultured with MC38 cells at an effector-to-target ratio of 10:1 for 24 h. Killing efficiency was assessed by flow cytometry using an Annexin V-FITC/PI double staining kit (BioLegend) to detect early and late apoptotic cells. For luciferase-labeled MC38 cells, tumor cell viability was indirectly measured by quantifying luminescence, reflecting the extent of CD8⁺ T cell-mediated killing. All experiments were performed in triplicate, and mean values were used for analysis.

### Nuclear-cytoplasmic fractionation and YAP1 nuclear localization detection

To investigate the regulation of Hippo pathway activity by CCL24/CCR3 signaling, nuclear-cytoplasmic fractionation was performed to assess YAP1 subcellular distribution. RAW264.7 cells were co-cultured with WT, CCL24^ko^, or mCCL24-supplemented MC38 cells for 48 h. Nuclear and cytoplasmic proteins were extracted using the NE-PER Nuclear and Cytoplasmic Extraction Kit (Thermo Fisher). Protein samples were quantified by BCA assay and subjected to Western blot (WB) analysis to detect YAP1 distribution in different cellular fractions, with Lamin B serves as the nuclear reference and GAPDH as the cytoplasmic reference. Images were analyzed for grayscale quantification using ImageJ software to evaluate changes in YAP1 nuclear localization.

### Immunofluorescence staining

To visualize YAP1 subcellular localization, immunofluorescence staining was performed on RAW264.7 cells co-cultured on slides. Cells were fixed for 15 min, permeabilized for 5 min, and blocked with 5% BSA for 1 h. Primary antibody against YAP1 (Cell Signaling Technology, 1:200 dilution) was incubated overnight at 4 ºC, followed by washing and incubation with Alexa Fluor 488-labeled secondary antibody for 1 h. Nuclei were stained with DAPI. Images were observed under a confocal microscope (Leica SP8), and nuclear and cytoplasmic fluorescence intensities were measured using ImageJ.

### Quantitative polymerase chain reaction (qPCR) analysis

To detect expression changes in YAP1 downstream target genes and related immunomodulatory genes, total RNA was extracted using TRIzol reagent (Invitrogen) and purified by centrifugation at 4 ºC adhering to the manufacturer's protocol. RNA concentration and integrity were verified using NanoDrop 2000 and agarose gel electrophoresis. Reverse transcription was conducted utilizing HiScript III RT SuperMix for qPCR (Vazyme). Real-time qPCR analysis was conducted using ChamQ SYBR qPCR Master Mix (Vazyme) on an ABI 7500 system, with amplification and melting curves strictly validated. Target genes included YAP1 pathway downstream genes (CTGF, CYR61, ANKRD1) and immunosuppression-related genes (Arg1, Mrc1, Chi3l3, Retnla), with primers synthesized by TSINGKE. Relative expression was calculated using the 2^-ΔΔCt^ method, with GAPDH as the reference gene.

### Molecular docking analysis

To develop potential pharmacological inhibitors of CCL24, molecular docking was performed using AutoDock Vina (v1.1.2). The three-dimensional protein structure of CCL24 was obtained from the AlphaFold Protein Structure Database (AF-Q9NPB8), and the small molecule structure was retrieved from PubChem (CID: 124423). Protein preprocessing included water removal, hydrogen addition, and Gasteiger charge assignment using AutoDockTools (v1.5.6) to generate pdbqt files. The docking grid box was set at 40 × 40 × 40 Å, centered on the N-terminal amino acid binding pocket of CCL24. After docking, the most stable conformation was selected based on binding affinity scores and visualized using PyMOL.

### Azoxymethane (AOM)/dextran sulfate sodium (DSS) stimulation for spontaneous CRC induction

To simulate chronic inflammation-driven colorectal tumorigenesis, a chemically induced mouse CRC model was established. Male C57BL/6J mice (SPF grade, 6-8 weeks old, Anburui.) were randomly assigned to groups of 6~8 mice each. The model was induced as follows: on day 1, AOM (Sigma, 10 mg/kg) dissolved in sterile PBS, with thorough mixing to ensure suspension uniformity, was intraperitoneally injected at an injection volume of 10 mL/kg. Starting on day 6, 2% dextran sulfate sodium (DSS, MP Biomedicals, molecular weight 36,000-50,000) was provided in drinking water for 7 days, followed by 14 days of regular drinking water, constituting one DSS cycle. Three DSS cycles were completed, with the entire experiment lasting approximately 8 weeks. The mouse body weight, water intake, and stool characteristics were monitored during the experiment. Mice exhibiting significant weight loss (> 20%), reduced activity, or hematochezia were euthanized early per ethical standards. At the end of week 8, mice were euthanized by cervical dislocation, and colons were harvested for histological, immunological, and molecular analyses.

### Construction and validation of CCL24 conditional knockout mice

To investigate the function of CCL24 in CRC development, intestinal epithelium-specific CCL24 knockout mice were generated. CCL24^fl/fl^ mice (prepared by Cyagen Biosciences) were crossed with Villin-Cre mice (purchased from Jackson Laboratory) to generate Villin-Cre; CCL24^fl/fl^ (CCL24^cko^) mice. Genotyping of F1 mice was performed by collecting tail tip tissue, extracting genomic DNA using a standard SDS-proteinase K lysis method, and amplifying CCL24^flox^ and Cre sequences with specific primers. PCR products were verified by agarose gel electrophoresis. CCL24^cko^ and CCL24^fl/fl^ control mice underwent AOM/DSS induction modeling at 8 weeks of age, with all mice processed in the same batch to avoid batch effects.

### *In vivo* intervention with CCL24 and CD8 neutralizing antibodies

After 8 weeks of AOM/DSS modeling, mice were divided into groups and subjected to intervention treatments. A CCL24 neutralizing antibody (CM101, MedChemExpress, designated as ACl24 for anti-CCL24) was administered via tail vein injection at 10 μg/kg once weekly, with an injection volume of 200 μL, for 3 weeks. Anti-CD8α antibody (BioXcell, Catalog no. BE0004-1, clone 53-6.7) was used to deplete CD8^+^ T cell activity, administered via tail vein injection at 200 μg per mouse every 5 days for a total of three injections. Tails were soaked in 42 ºC warm water for 2 min before injection to dilate blood vessels, ensuring injection success and reproducibility.

### Subcutaneous tumor and metastasis model establishment and treatment regimens

WT or CCL24^ko^ MC38 cells were injected into BALB/c nude mice or C57BL/6 mice subcutaneously to generate tumors. In brief, approximately 1 × 10^6^ WT or CCL24^ko^ MC38 cells suspended in 100 μL PBS were injected into the right flank of C57BL/6 mice. The tumor volume was determined once a week with the following formula: length × width^2^ × π/6. After 4 weeks, the mice were euthanized, and the tumor tissues were excised, weighed, and prepared for subsequent experiments.

Furthermore, to investigate the* in vivo* antitumor effects of Gracillin with other anti-cancer treatments, 1 × 10^6^ WT MC38 cells suspended in 100 μL PBS were injected into the right flank of C57BL/6 mice. Once tumor volumes reached approximately 100 mm^3^, mice were randomized into groups receiving corn oil (control), Gracillin (20 mg/kg, i.p., every other day), 5-fluorouracil (5-FU, 30 mg/kg, i.p., twice weekly), or the combination therapy of Gracillin and 5-FU. Treatment continued for 10 days, after which tumor volume and wet weight were recorded.

For the metastasis model, 1 × 10^6^ luciferase-labeled MC38 cells were injected via the mouse tail vein. Treatment began on day 3 post-modeling, with administration of monoclonal antibody (mAb) of PD-1 (BioXcell, BE0146, clone RMP1-14, 200 μg per mouse, i.p., every 3 days) and Gracillin (20 mg/kg) alone or in combination for 2 weeks. On day 21, tumor burden was monitored using small-animal *in vivo* fluorescence imaging (IVIS Lumina III, PerkinElmer). After euthanasia, lung and liver tissues were harvested for counting metastatic nodules and flow cytometric analysis.

### T cell function restoration experiment

To investigate the impact of CCL24 on T cell-dependent immune responses, T cell function was reconstituted in immunocompromised nude mice. Thymus tissue from BALB/c mice was processed into a single-cell suspension, and activated CD8^+^ T cells were prepared as described above. These T cells (5 × 10^6^) were resuspended in PBS and injected via the tail vein into BALB/c nude mice previously inoculated with MC38 cells (WT or CCL24^ko^), with an injection volume of 200 μL per mouse. Tumor growth was monitored post-injection, and samples were collected on day 28 to assess the impact of T cell function restoration on tumor growth in different cell lines.

### Tumor burden assessment and histological analysis

After euthanasia, colons were carefully excised, gently rinsed with PBS, and photographed on a black background (Nikon D750). Tumor nodules were measured using a vernier caliper, with nodules >1 mm in diameter counted as valid tumors. Data were plotted using GraphPad Prism. Colon tissue segments were divided for analysis: one portion was fixed in 4% paraformaldehyde, paraffin-embedded, and sectioned (4 μm) for hematoxylin-eosin (HE) staining and immunohistochemistry (IHC); another portion was digested with collagenase/hyaluronidase to prepare single-cell suspensions for flow cytometry. IHC was performed using anti-Cleaved-Caspase-3 (C-Cas-3) and anti-CCR3 antibodies (Cell Signaling Technology), with secondary antibodies visualized using a DAB detection system (ZSGB-BIO). Stained sections were scanned, and integrated optical density was quantified using ImageJ.

### Chromatin-immunoprecipitation (ChIP)-seq and ChIP-qPCR analysis

To elucidate the direct regulatory role of YAP1 in immunosuppressive polarization-related genes in TAMs, ChIP experiments were conducted on CCR3^+^ TAMs sorted from tumor tissues of CCL24^fl/fl^ and CCL24^cko^ mice. Cells were crosslinked with 1% formaldehyde for 10 min, followed by quenching. A ChIP kit (Millipore) was used following the manufacturer's guidelines, with anti-YAP1 (Cell Signaling Technology) to enrich YAP1-bound chromatin fragments. ChIP-seq libraries were constructed and sequenced on the Illumina NovaSeq platform using paired-end sequencing. Raw data underwent quality control with FastQC, alignment to the mouse reference genome (mm10) using Bowtie2, peak calling with MACS2, and annotation and functional enrichment analysis with HOMER. Results were visualized using IGV software. ChIP-qPCR was performed to validate YAP1 binding abundance at the transcription start site (TSS) of genes such as Arg1, Mrc1, Chi3l3, and Retnla, with amplified fragments covering core promoter sequences. Results were expressed as relative enrichment fold changes, with IgG as the negative control.

### YAP1 functional intervention experiments

To verify the functional role of YAP1 in immunosuppressive polarization of TAMs, RAW264.7 cells were treated with the LATS1 kinase-specific inhibitor TRULI (10 μM, MedChemExpress) to promote YAP1 dephosphorylation and nuclear activation. TRULI was added to the co-culture system with CCL24^ko^ MC38 cells for 48 h, followed by cell collection. Flow cytometry was used to detect the positive expression ratios of Arg1 and Mrc1 to assess changes in TAM polarization status. Cell staining was performed using antibodies against F4/80, Arg1, and Mrc1 (BioLegend), with analysis conducted using FlowJo software. A DMSO-treated group served as the control.

### YAP1 modulation and T cell functional assays

To assess the direct impact of macrophage YAP1 activity on T cell function, RAW264.7 cells were subjected to gain- or loss-of-function interventions. For gain-of-function, cells were treated with the LATS1/2 inhibitor TRULI (10 μM) to induce constitutive YAP1 nuclear localization. For loss-of-function, cells were transfected with YAP1-specific siRNA (siYAP1) or treated with the YAP1 inhibitor Verteporfin (2 μM). Following modulation, macrophages were co-cultured with activated CD8+ T cells (ratio 1:5) for 24 h. T cell function was subsequently analyzed by flow cytometry for intracellular Granzyme B (GZMB) and TNFA expression, as described in the cytokine detection section.

### The Cancer Genome Atlas (TCGA) and Tumor Immune Dysfunction and Exclusion (TIDE) data analysis

To explore the correlation between CCL24 expression, immune status, and prognosis, transcriptomic expression data (FPKM format) and clinical follow-up information from the TCGA-COAD project were downloaded. Data preprocessing and standardization were performed in R (v4.2.2) using the limma and survival packages to analyze the relationship between CCL24 expression and survival. Kaplan-Meier survival curves were generated using survminer. Immune infiltration correlations were assessed by analyzing Pearson correlations between CCL24 and T cell dysfunction scores, cytotoxic T lymphocyte (CTL) infiltration scores, and TAM marker genes. The TIDE database was used to predict CCL24's potential for immune escape, including T cell dysfunction and exclusion scores, and to evaluate its predictive capacity for immunotherapy response.

### Single-cell transcriptomic analysis

To investigate the impact of CCL24 knockout on the TME, single-cell RNA sequencing (scRNA-seq) was performed on colon tumor tissues from CCL24^cko^ and CCL24^fl/fl^ mice. Tissues were enzymatically dissociated with collagenase/hyaluronidase and filtered through a 40 μm mesh to obtain single-cell suspensions. Libraries were constructed using the 10x Genomics Chromium platform (3' v3 chemistry) and sequenced on the NovaSeq 6000. Raw data were processed with Cell Ranger (v6.1.2) for quality control and alignment, followed by analysis in Seurat (v4.3.0) for linear normalization, PCA dimensionality reduction, UMAP clustering, and cell annotation. Cell types were identified based on classic markers (e.g., CD3D, CD8A, CD68, LY6G). Differential analysis (FindMarkers) was used to compare the proportions and transcriptional differences of TAMs, CD8^+^ T cells, and other immune cell populations between CCL24^cko^ and control groups. Results were visualized using DotPlot and VlnPlot and used for downstream functional annotation.

### Cell-cell communication analysis (CellChat)

To explore CCL24-mediated cell-cell communication pathways in the TME, the CellChat package (v1.6.1) was applied to single-cell data. Seurat objects were converted to CellChat format, with cell subsets defined as nodes. Ligand-receptor pair communication probabilities were calculated using the CellChatDB.mouse database, with the inference method set to “trimean” and a minimum cell threshold of > 10.

### RNA-sequencing (RNA-seq) and Gene Set Enrichment Analysis (GSEA)

To elucidate transcriptional changes in TAMs under CCL24 regulation, CD11b^+^F4/80^+^CCR3^+^ TAMs were sorted from colon tumor tissues of CCL24^fl/fl^ and CCL24^cko^ mice. Total RNA was extracted, libraries were constructed, and high-throughput sequencing was performed on the Illumina NovaSeq platform. Data quality control was conducted with FastQC, alignment to the mm10 reference genome with Hisat2, and gene counting with HTSeq. Differential expression analysis was performed using DESeq2 (v1.36.0), with a threshold of FDR < 0.05 and |log2FC| > 1. GSEA was conducted using ClusterProfiler (v4.6.0) with gene sets from MSigDB (v7.5.1) C2 and C5 databases, focusing on the Hippo signaling pathway (hsa04390).

### ChIP-seq data analysis

To investigate the epigenetic mechanisms of YAP1-mediated regulation of target genes in immunosuppressive TAMs, YAP1 ChIP-seq analysis was performed. CCR3^+^ TAMs were processed with anti-YAP1 antibody (Cell Signaling Technology) for ChIP to enrich chromatin fragments, followed by library construction and sequencing on the Illumina NovaSeq platform. Reads were cleaned using fastp, aligned to the mm10 genome with Bowtie2, and peak calling was performed with MACS2 (v2.2.7.1) using parameters --nomodel --qvalue 0.01. Peaks were annotated with ChIPseeker, and motif enrichment was analyzed with HOMER. Binding peaks were intersected with RNA-seq differentially expressed genes (DEGs) to predict YAP1 direct regulatory targets.

### Surface plasmon resonance (SPR) assay

The binding affinity between Gracillin and CCL24 was measured using a Biacore T200 system (GE Healthcare). Recombinant human CCL24 protein was immobilized on a CM5 sensor chip using standard amine coupling. Gracillin was diluted in running buffer (PBS with 0.05% Tween-20 and 1% DMSO) to various concentrations (3.125, 6.25, 12.5, 25, and 50 μM) and injected over the chip surface at a flow rate of 30 μL/min. The association and dissociation phases were monitored for 120 seconds each. The equilibrium dissociation constant (K_D_) was calculated using the steady-state affinity fitting model in the Biacore evaluation software.

### Statistical analysis

All statistical analyses were performed using GraphPad Prism 9.0. Experiments were generally repeated at least six times, with data expressed as mean ± standard deviation. Group comparisons were conducted using an unpaired two-tailed Student's t-test or one-/two-way ANOVA with Tukey's post-hoc test, as appropriate. Animal study groups included six subjects each. Statistical significance was defined as p < 0.05.

## Results

### CCL24 blockade reduces CRC initiation and progression *in vivo*

**Figure 1:**
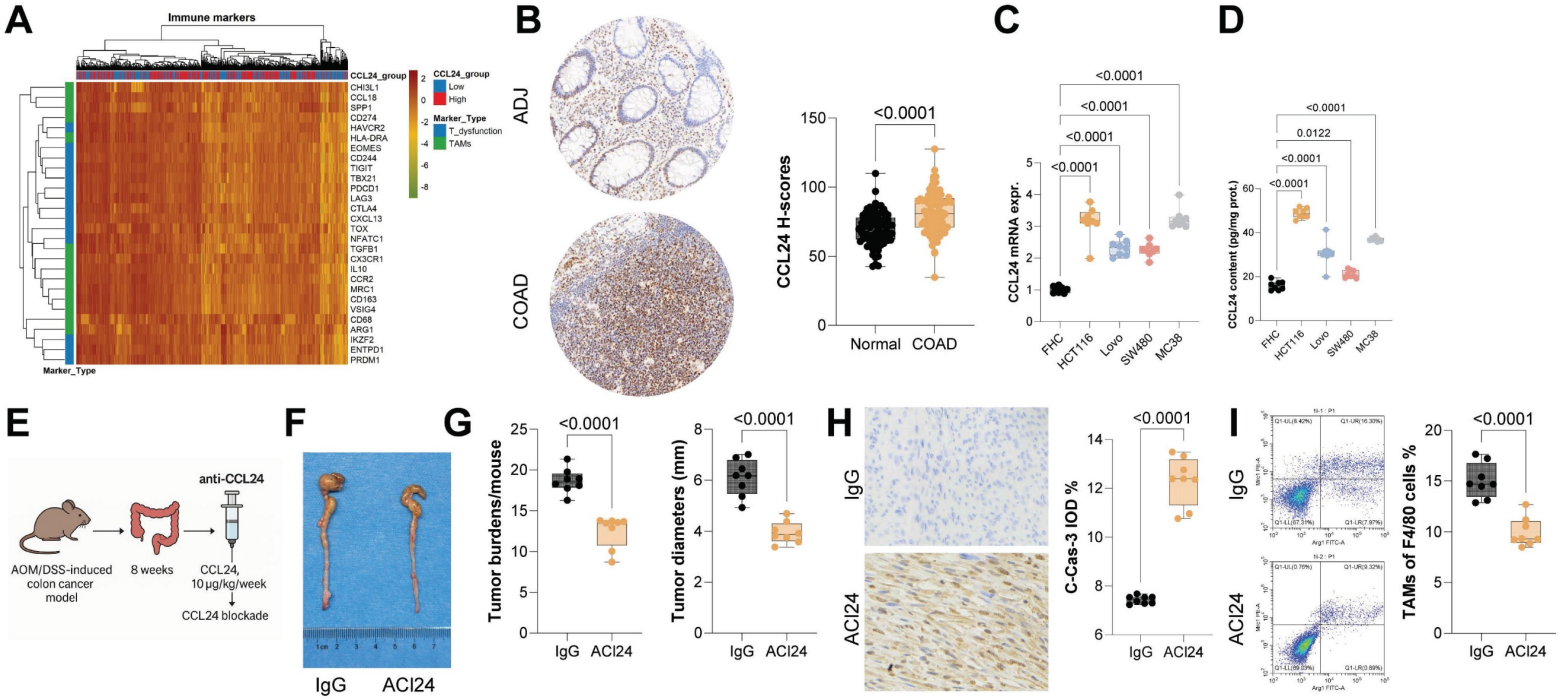
CCL24 blockade reduces CRC incidence and progression *in vivo*. A, Correlation analysis of CCL24 expression with T cell dysfunction and TAM-related marker genes in the TCGA-COAD dataset. B, TMA analysis of CCL24 staining intensity in tumor and adjacent tissues from 84 CRC patients. C-D, qPCR and ELISA detection of CCL24 mRNA and secretion levels in colon epithelial cells (FHC) and CRC cell lines (HCT116, Lovo, SW480, MC38). E, a primary CRC model was established in C57BL/6 mice through AOM/DSS induction, followed by tail vein injection of anti-CCL24 (ACl24, 10 μg/kg/week) after 8 weeks for CCL24 blockade. F, Gross images of mouse colons with primary tumors. G, Tumor number and diameter in mouse colons. H, IHC detection of C-Cas-3 positive staining in tumor tissues. I, Flow cytometry analysis of Arg1^+^Mrc1^+^ TAMs in mouse colon tissues. TMA includes 84 pairs of tissue samples. Cell experiments repeated 6-8 times. Animal experiments include 6-8 mice per group. Data are presented as bars and dots. *p* < 0.05 was considered statistically significant.

To investigate the roles of CCL24 in CRC, we first queried the TCGA-COAD database, identifying a link between high CCL24 expression and poor prognosis in patients (Fig. S1A). Data from this system also suggest that CCL24 levels are correlated positively with T cell dysfunction and negatively with CTL infiltration in CRC patients (Fig. S1B-C). Consistently, high CCL24 expression was found to be linked to increased T cell exhaustion- and TAM-related markers in TCGA-COAD dataset (Fig. 1A). To verify the bioinformatics trends in clinical samples, we analyzed TMA data including 84 pairs of tumor and normal tissue samples from CRC patients. Notably, increased CCL24 H-scores were detected in CRC tissues versus the normal counterparts (Fig. 1B). Those with high CCL24 expression exhibited advanced clinical staging and poor prognosis (Fig. S1D-E), which are consistent with the bioinformatics data. Additionally, it was noted that the mRNA expression of CCL24 in FHC cells, as well as the CCL24 secretion in the cell culture supernatant, was significantly lower than that by CRC cell lines (Fig. 1C-D).

To examine the precise function of CCL24 in CRC progression, a primary CRC model was established in C57BL/6 mice through AOM/DSS induction, followed by tail vein injection of anti-CCL24 (ACl24, 10 μg/kg/week) for CCL24 blockade after 8 weeks (Fig. 1E). Notably, the ACl24 treatment markedly reduced both the number and size of tumors in mouse colon tissues (Fig. 1F-G), along with increased positive staining of C-Cas-3 in tumor tissues (Fig. 1H). The number of immunosuppressive TAMs (F4/80⁺Arg1⁺/Mrc1⁺) in tumor tissues was substantially reduced after CCL24 blockade (Fig. 1I).

### CCL24 knockout reduces CRC progression by altering the TME

**Figure 2:**
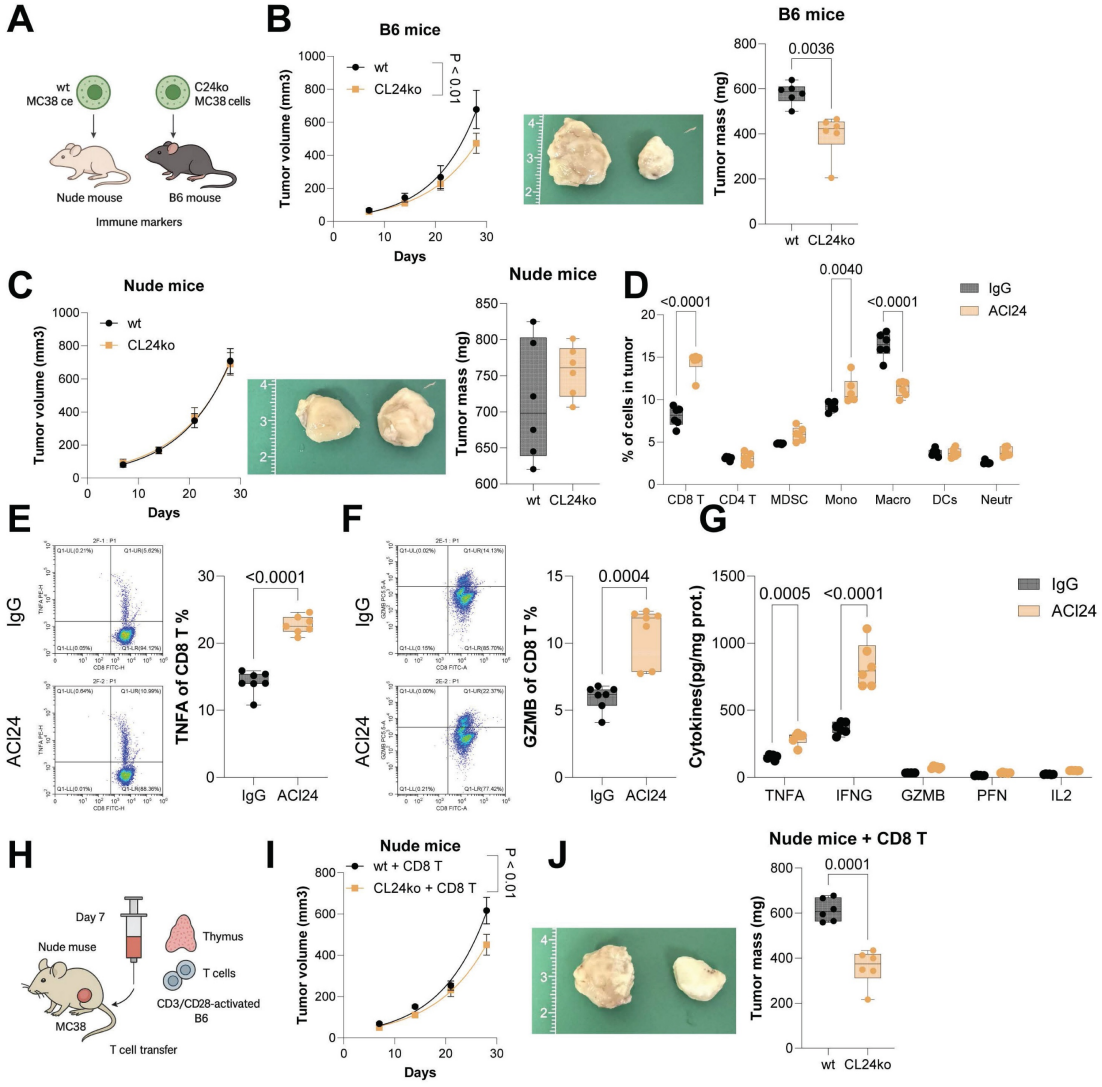
CCL24 knockout reduces CRC progression by altering the TME. WT or CCL24^ko^ MC38 cells were injected into immunocompetent C57BL/6 mice or immunocompromised nude mice subcutaneously. B-C, Growth rates of tumors formed by WT or CCL24^ko^ cells in C57BL/6 (B) and nude (C) mice. D, Flow cytometry analysis of immune cell populations in mouse primary tumor tissues after ACl24 treatment: CD8^+^ T (CD3^+^CD45^+^CD8^+^), CD4^+^ T (CD3^+^CD45^+^CD4^+^), myeloid-derived suppressor cells (CD11b^+^Gr1^+^), monocytes (CD11b^+^Ly6C^+^), macrophages (CD11b^+^F4/80^+^), DCs (CD11c^+^MHCII^+^), and neutrophils (CD11b^+^Ly6G^+^). E-F, Populations of TNFA^+^ and GZMB^+^ CD8^+^ T cells in AOM/DSS-induced tumors in mice after ACl24 treatment determined using flow cytometry. G, Serum levels of cytotoxic cytokines (TNFA, IFNG, GZMB, PFN, and IL2) in mice after ACl24 treatment determined using ELISA kits. H, Nude mice inoculated with WT or CCL24^ko^ MC38 cells were further injected with CD3/CD28-activated CD8^+^ T cells derived from C57BL/6 mouse thymus 7 days post-inoculation via the tail vein. I-J, Analysis of MC38 cell growth rate and tumor weight in nude mice. Cell experiments repeated 6-8 times. Animal experiments include 6-8 mice per group. Data are presented as bars and dots. *p* < 0.05 was considered statistically significant.

CCL24^ko^ HCT116 and MC38 cell lines were generated using CRISPR/Cas9 technique (Fig. S2A-B). Proliferation or invasion of these two cell lines* in vitro* was not significantly restricted following CCL24 knockout (Fig. S2C-D). However, compared to WT cells, the tumorigenic capacity of CCL24^ko^ MC38 cells was substantially in C57BL/6 mice in subcutaneous models (Fig. 2A-B). By contrast, the CCL24 knockout did not affect tumorigenesis of MC38 cells in immunocompromised nude mice (Fig. 2C). This finding suggested that CCL24 might influence CRC development though the adaptive immune system.

To further investigate whether CCL24 affects CRC progression by modulating immune responses, immune cell differences in colon tumor tissues from ACl24-treated mice were analyzed. A marked increase was detected in CD8^+^ T cell infiltration (Fig. 2D). Similarly, the number of CD8^+^ T cells in tumors formed by CCL24^ko^ MC38 cells in C57BL/6 mice was markedly elevated (Fig. S2E). Furthermore, flow cytometry analysis revealed a substantial increase in TNFA^+^ and GZMB^+^ CD8^+^ T cells in primary tumor tissues from ACl24-treated mice (Fig. 2E-F). Serum levels of cytotoxic cytokines (TNFA, IFNG, GZMB, PFN, and IL2) were also markedly elevated following ACl24 treatment (Fig. 2G).

To further confirm whether CCL24 influences CD8^+^ T cell function by altering the TME, nude mice inoculated with MC38 cells were injected with CD3/CD28-activated CD8^+^ T cells derived from C57BL/6 mouse thymus 7 days post-inoculation via the tail vein (Fig. 2H). Interestingly, compared to WT cells, growth of CCL24^ko^ cells in nude mice was markedly reduced upon CD8^+^ T cell injection (Fig. 2I-J). In contrast, intratumoral injection of mouse recombinant CCL24 protein (mCCL24) in C57BL/6 mice markedly promoted growth of tumors formed by CCL24^ko^ MC38 cells (Fig. S3A-C), with a notable reduction in CD8^+^ T cell numbers in tumor tissue (Fig. S3D).

### CCL24 knockout in colon epithelium reduces AOM/DSS-induced tumorigenesis and enhances CD8^+^ T cell function in mice

**Figure 3:**
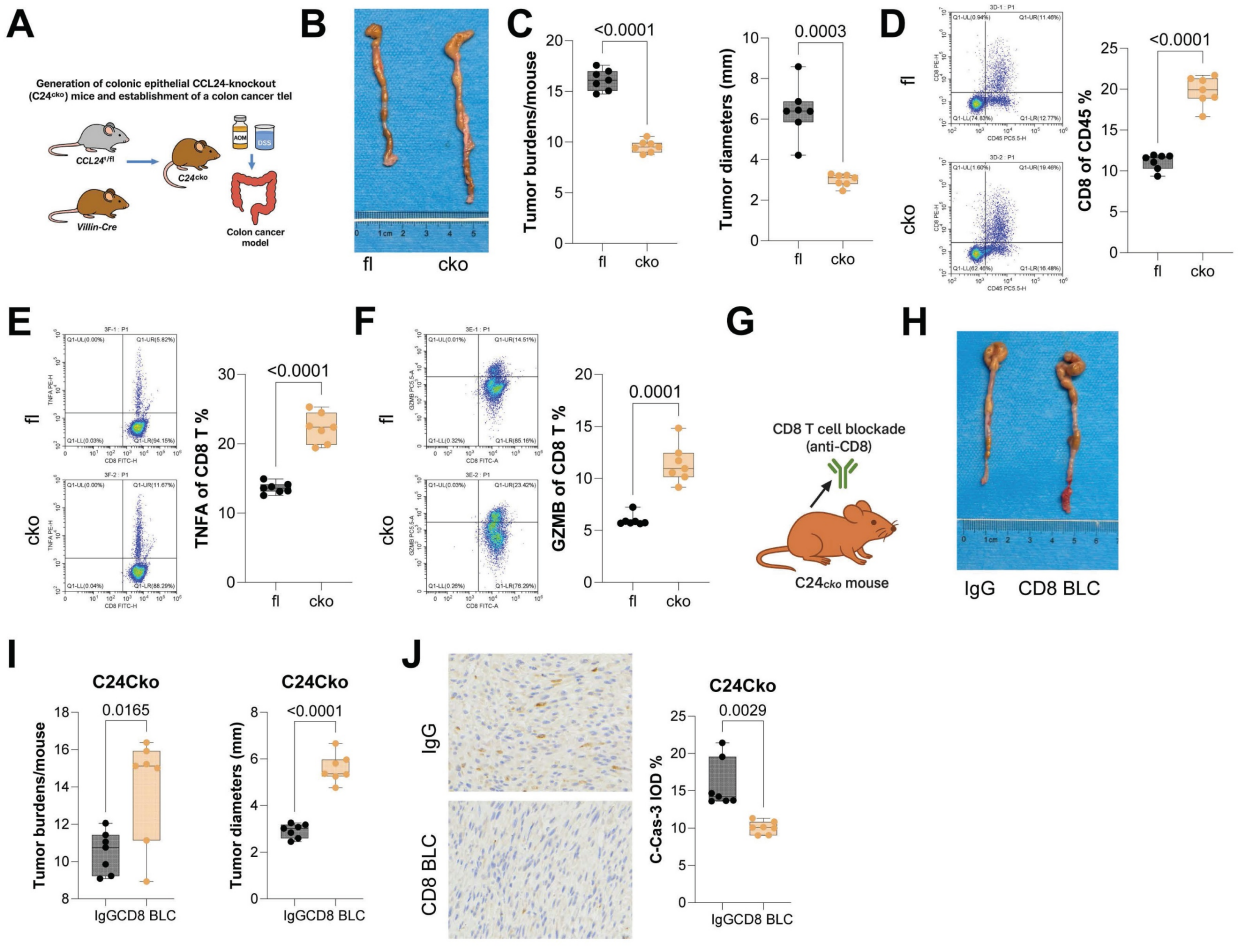
CCL24 knockout in colon epithelium reduces AOM/DSS-induced tumorigenesis and enhances CD8^+^ T cell function in mice. A, CCL24^fl/fl^ and Villin-Cre mice were allowed to mate to generate mice with colon epithelium-specific CCL24 conditional knockout (C24^cko^), followed by AOM/DSS challenge to induce colorectal tumorigenesis. B, Gross images of mouse colons. C, Tumor number and diameter in mouse colons. D-F, Populations of total CD8^+^ T cells (D), TNFA^+^ (E), and GZMB^+^ (F) CD8^+^ T cells in mouse tumor tissues. G, Anti-CD8 antibody treatment for CD8^+^ T cell depletion in AOM/DSS-challenged C24^cko^ mice after 8 weeks. H, Gross images of mouse colons. I, Tumor number and diameter in mouse colons. J, IHC detection of C-Cas-3 staining in tumor tissues. Each group includes 6-8 mice. Data are presented as bars and dots. *p* < 0.05 was considered statistically significant.

CCL24^fl/fl^ and Villin-Cre mice were allowed to mate to generate mice with colon epithelium-specific CCL24 conditional knockout (CCL24^cko^), followed by AOM/DSS challenge to induce CRC genesis (Fig. 3A). Tumor number and size in colon tissue of CCL24^cko^ mice were significantly reduced compared to those in CCL24^fl/fl^ controls (Fig. 3B-C). Further investigation focused on changes in CD8^+^ T cell infiltration in tumor tissue. Consistent with ACl24 results, the total number of CD8^+^ T cells, as well as TNFA^+^ and GZMB^+^ CD8^+^ T cells, was significantly increased in CCL24^cko^ mouse tumors (Fig. 3D-F). Furthermore, to examine the dependence of CD8^+^ T cells in CCL24^cko^-associated events, CCL24^cko^ mice with AOM/DSS challenge were additionally treated with the anti-CD8 antibody (Fig. 3G). Following CD8^+^ T cell blockade, tumor burden in colon tissue was significantly restored (Fig. 3H-I), and C-Cas-3 staining intensity in tumor tissue was markedly reduced (Fig. 3J).

Furthermore, to determine whether CCL24 directly affects T cell activity, CD8^+^ T cells were sorted from mouse thymocytes, activated with CD3/CD28 antibodies, sorted, and co-cultured with WT or CCL24^ko^ MC38 cells* in vitro* (Fig. S4A). Co-culturing with MC38 cells substantially reduced the populations of TNFA^+^ and GZMB^+^ CD8^+^ T cells; however, these populations were not significantly altered regardless of CCL24 knockout or not in MC38 cells (Fig. S4B-C). This lack of direct effect on T cells is a vital control finding: it confirms that CCL24 does not directly signal to CD8+ T cells to induce exhaustion. Instead, it suggests that the immunosuppressive function of CCL24 is strictly dependent on (an) intermediate cell type (s) to bridge the interaction between the tumor and the adaptive immune system.

### CCL24^+^ tumor cells recruit CCR3^+^ TAMs to augment immunosuppression

**Figure 4:**
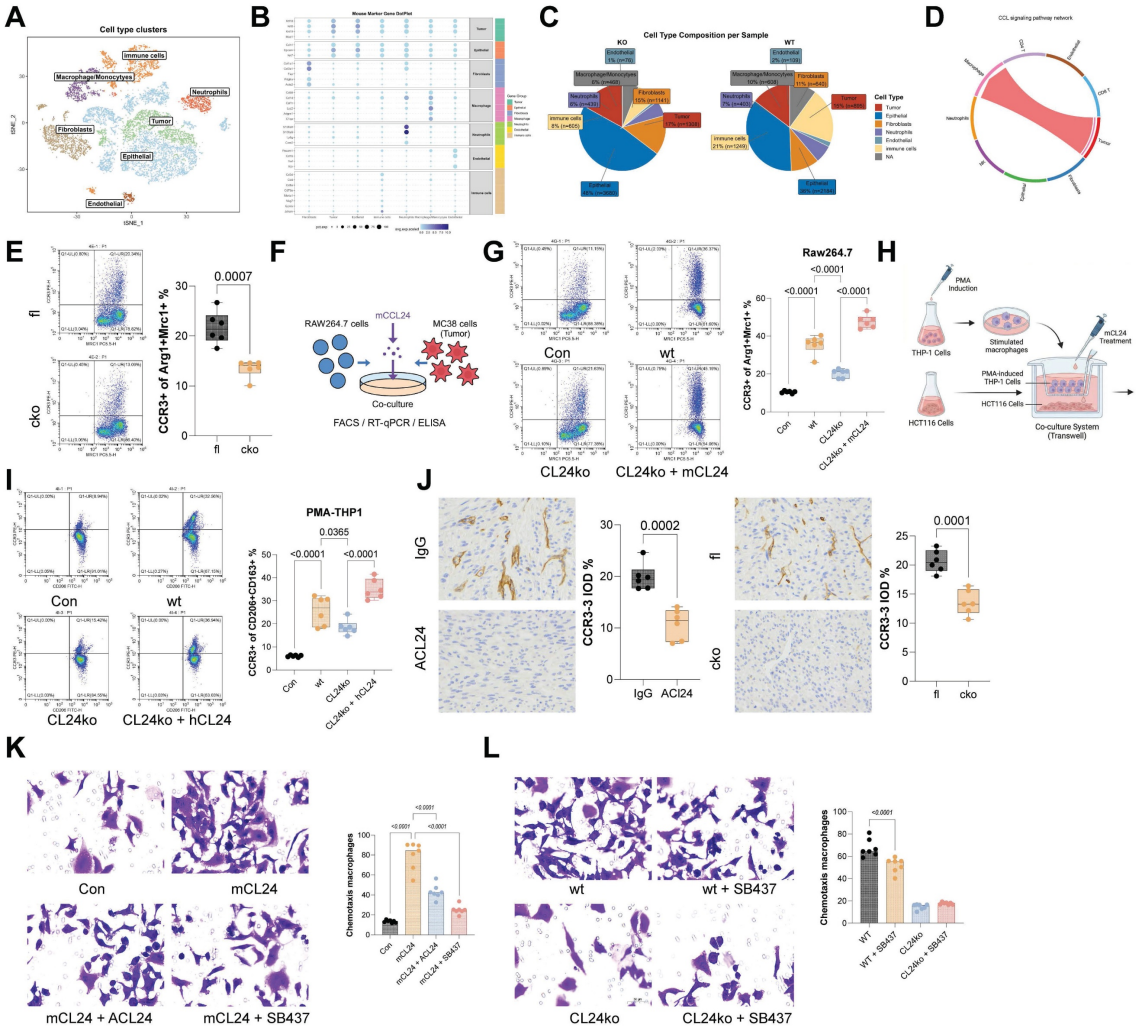
CCL24+ tumor cells recruit CCR3+ TAMs to augment immunosuppression. A, UMAP visualization of cell distribution in mouse colon tumor tissue. B, Markers for various cell types. C, Proportions of various cell types. D, CellChat analysis of tumor cell-macrophage interaction in mouse colon tissues. E, Number of CCR3^+^ TAMs in the colon tissues of C24^cko^ and CCL24^fl/fl^ mice with AOM/DSS induction determined using flow cytometry. F, Mouse RAW264.7 macrophages were co-cultured with WT or CL24^ko^ MC38 cells, followed by the addition of mCCL24. G, Number of CCR3^+^Arg1^+^Mrc1^+^ RAW264.7 cells in different settings determined using flow cytometry. H, Co-culture of human CRC cells (HCT116) with PMA-induced WT or CL24^ko^ THP-1 cells, with mCCL24 supplementation. I, Population of CCR3^+^CD206^+^CD163^+^ in THP-1 cells determined using flow cytometry. Positive staining for CCR3 in colon tumor tissue from ACl24-treated mice or C24^cko^ mice with AOM/DSS induction. Cell experiments repeated 6-8 times. Animal experiments include 6-8 mice per group. Data are presented as bars and dots. *p* < 0.05 was considered statistically significant.

As a chemokine, CCL24 primarily functions in recruiting immune cells, with CCR3 known as its primary receptor, which is mainly expressed on eosinophils, basophils, Th2 cells, and TAMs as well 24. To study further the primary immune cell population affected in CCL24 in this context, scRNA-seq analysis was performed to analyze cell population changes between CCL24^cko^ and CCL24^fl/fl^ mice. The results revealed a significant reduction in macrophage proportion in CCL24^cko^ mice, with increased CD8^+^ T cell numbers but no significant differences in eosinophils (Fig. 4A-C). CellChat analysis further verified that tumor cells interact with macrophages via the CCL24-CCR3 axis (Fig. 4D, Fig. S5). Notably, the number of CCR3^+^ TAMs was found to be reduced in CCL24^cko^ mice (Fig. 4E). These results indicated that CCL24 secreted by CRC cells might bind to CCR3 on macrophages, promoting TAM polarization and altering the TME.

To test this finding, mouse RAW264.7 macrophages were co-cultured with WT or CCL24^ko^ MC38 cells (Fig. 4F). Co-culturing with MC38 cells significantly increased the proportion of Arg1^+^Mrc1^+^ RAW264.7 cells, while this trend was partly diminished by the CCL24 knockout. Furthermore, supplementation of mCCL24 to the CCL24^ko^-RAW264.7 co-culture system restored the population of immunosuppressive macrophages (Fig. 4G). Parallel trends were observed in another co-culture system of human CRC cells (HCT116) and PMA-induced THP-1 cells (Fig. 4H-I). In AOM/DSS-induced primary tumor models, the positive staining for CCR3 was found to be reduced in both ACl24-treated mice and CCL24^cko^ mice (Fig. 4J). These results indicated that CCL24 secreted by CRC cells functions as a key chemoattractant for macrophages. To confirm the direct recruitment of TAMs by CCL24, we performed *in vitro* Transwell migration assays. The addition of mCCL24 significantly increased the migration of RAW264.7 macrophages compared to the control (Fig. 4K). Notably, pre-treatment of macrophages with the selective CCR3 antagonist SB-328437, or the addition of a CCL24-neutralizing antibody, almost completely abolished mCCL24-induced migration (Fig. 4K). Furthermore, CM from WT MC38 cells induced robust macrophage migration, whereas CM from CCL24^ko^ MC38 cells failed to recruit macrophages effectively. The addition of the CCR3 inhibitor to the WT CM reduced migration to levels comparable to the CCL24^ko^ group, confirming that CCL24 is the primary CCR3 ligand responsible for macrophage recruitment in this context (Fig. 4L).

### The CCL24-CCR3 axis promotes immunosuppressive skewing of TAMs by activating the Hippo pathway

**Figure 5:**
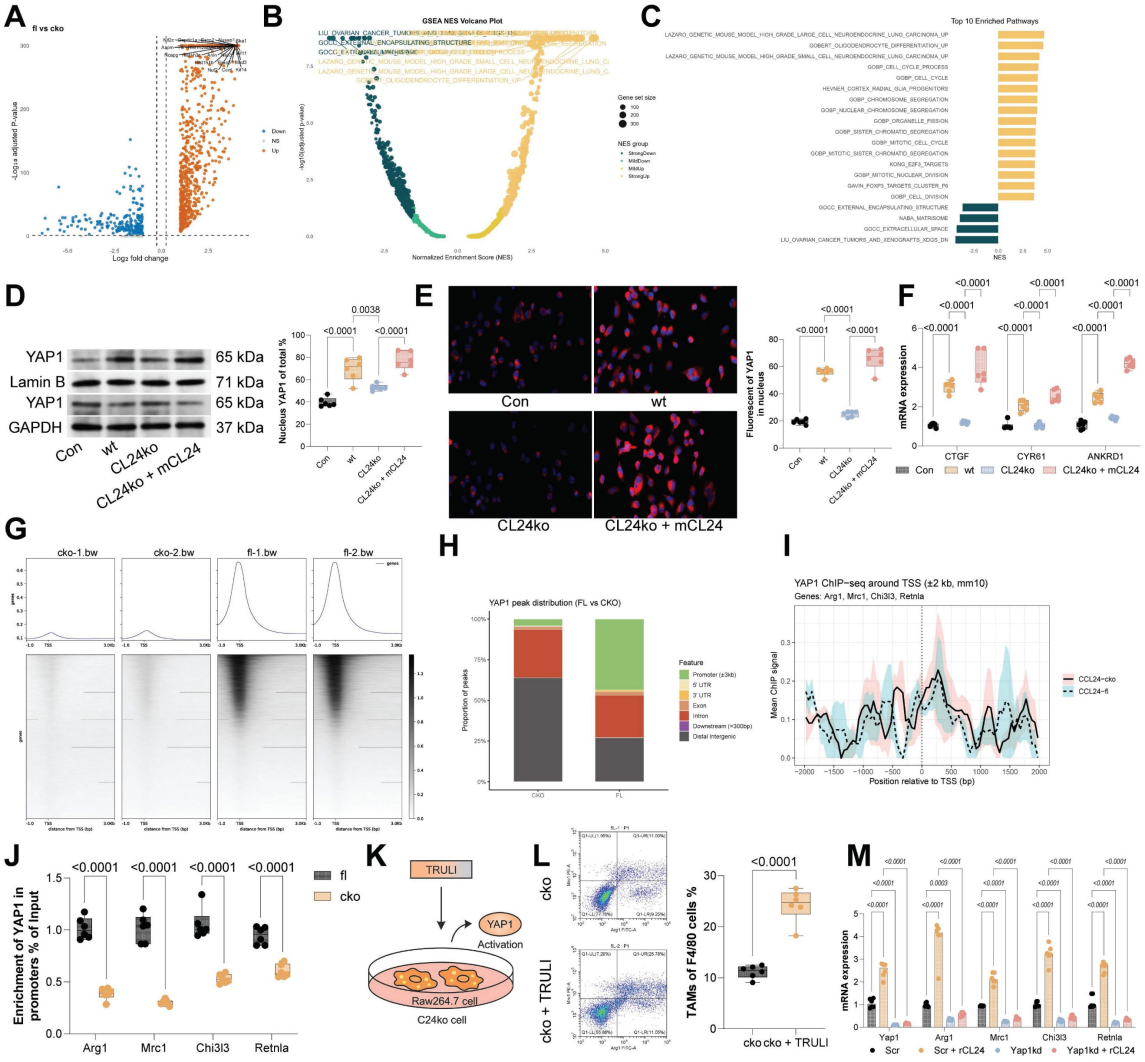
The CCL24-CCR3 axis promotes immunosuppressive skewing of TAMs by activating the Hippo pathway. A-B, Volcano plots and heatmaps of DEGs between CCR3^+^ and CCR3⁻ TAMs from CCL24^fl/fl^ and CCL24^cko^ mice identified using RNA-seq analysis. C, GSEA of DEGs. D, Nuclear-cytoplasmic component separation and WB analysis to detect YAP1 sub-cellular localization in RAW264.7 cells co-cultured with MC38 cells, with Lamin B as the nuclear marker and GAPDH as the cytoplasmic marker. E, Immunofluorescence detection of YAP1 nuclear accumulation in RAW264.7 cells co-cultured with MC38 cells (Red fluorescence indicates YAP1 protein; Blue fluorescence indicates DAPI nuclear staining). F, qPCR analysis of CTGF, CYR61, and ANKRD1 mRNA expression in RAW264.7 cells co-cultured with MC38 cells. G-H, ChIP-seq analysis of YAP1 genomic distribution. I, IGV visualization of YAP1 binding abundance at TSS regions of Arg1, Mrc1 (CD206), Chi3l3 (Ym1), and Retnla. J, ChIP-qPCR detection of YAP1 binding to promoter segments of Arg1, Mrc1 (CD206), Chi3l3 (Ym1), and Retnla using anti-YAP1 antibody. K, Treatment of RAW264.7 cells co-cultured with CCL24ko MC38 cells with LATS1-specific inhibitor TRULI to promote YAP1 activation. L, Population of Arg1^+^/Mrc1^+^ RAW264.7 cells determined using flow cytometry. M, macrophages were transfected with siNC or siYAP1 and stimulated with mCCL24, followed by detection of Arg1 and Mrc1 mRNA by qPCR analysis. Cell experiments repeated 6-8 times. Animal experiments include 6-8 mice per group. Data are presented as bars and dots. *p* < 0.05 was considered statistically significant.

To investigate the downstream molecular mechanisms involved in TAM polarization, RNA-seq analysis was performed on DEGs between CCR3^+^ and CCR3⁻ TAMs from CCL24^fl/fl^ and CCL24^cko^ mice, identifying 329 genes (Fig. 5A-B). GSEA of these genes indicated that the Hippo signaling pathway had the highest normalized enrichment score (Fig. 5C). To analyze the activation of this pathway, we analyzed nuclear translocation of YAP1 in RAW264.7 cells. Notably, co-culturing with WT MC38 cells substantially promoted YAP1 nuclear translocation in RAW264.7 cells, which was reduced upon CCL24 knockout. However, supplementation of mCCL24 to the culture system restored the nuclear accumulation of YAP1 (Fig. 5D-E). Parallel trends were observed in the expression of key YAP1 downstream genes (CTGF, CYR61, and ANKRD1) in RAW264.7 cells (Fig. 5F). To delve into YAP1's role in TAM polarization, ChIP-seq was performed using anti-YAP1 antibody, revealing significantly higher YAP1 binding to TSS regions in CCL24^fl/fl^ TAMs compared to that in CCL24^cko^ TAMs (Fig. 5G-H). Binding to TSS regions of Arg1, Mrc1 (CD206), Chi3l3 (Ym1), and Retnla was also significantly reduced in CCL24^cko^ TAMs (Fig. 5I). Consistently, ChIP-qPCR assays validated the reduced YAP1 binding to TSS regions of these genes (Fig. 5J). Additionally, RAW264.7 cells co-cultured with CCL24^ko^ MC38 cells were treated with the LATS1-specific inhibitor TRULI to promote YAP1 activation (Fig. 5K). Notably, the TRULI treatment markedly restored immunosuppressive polarization of RAW264.7 cells (Fig. 5L). To further verify the necessity of YAP1 in this process, we performed loss-of-function experiments using YAP1-specific siRNA. In macrophages transfected with control siRNA, CCL24 stimulation induced robust upregulation of Arg1 and Mrc1. However, this induction was significantly blunted in YAP1-knockdown cells (Fig. 5M). These combined gain- and loss-of-function results confirm that YAP1 acts as the critical downstream effector translating CCL24-CCR3 signaling into an immunosuppressive gene signature.

### CCR3^+^ TAMs show stronger suppressive effects on CD8^+^ T cells over CCR3⁻ TAMs

**Figure 6:**
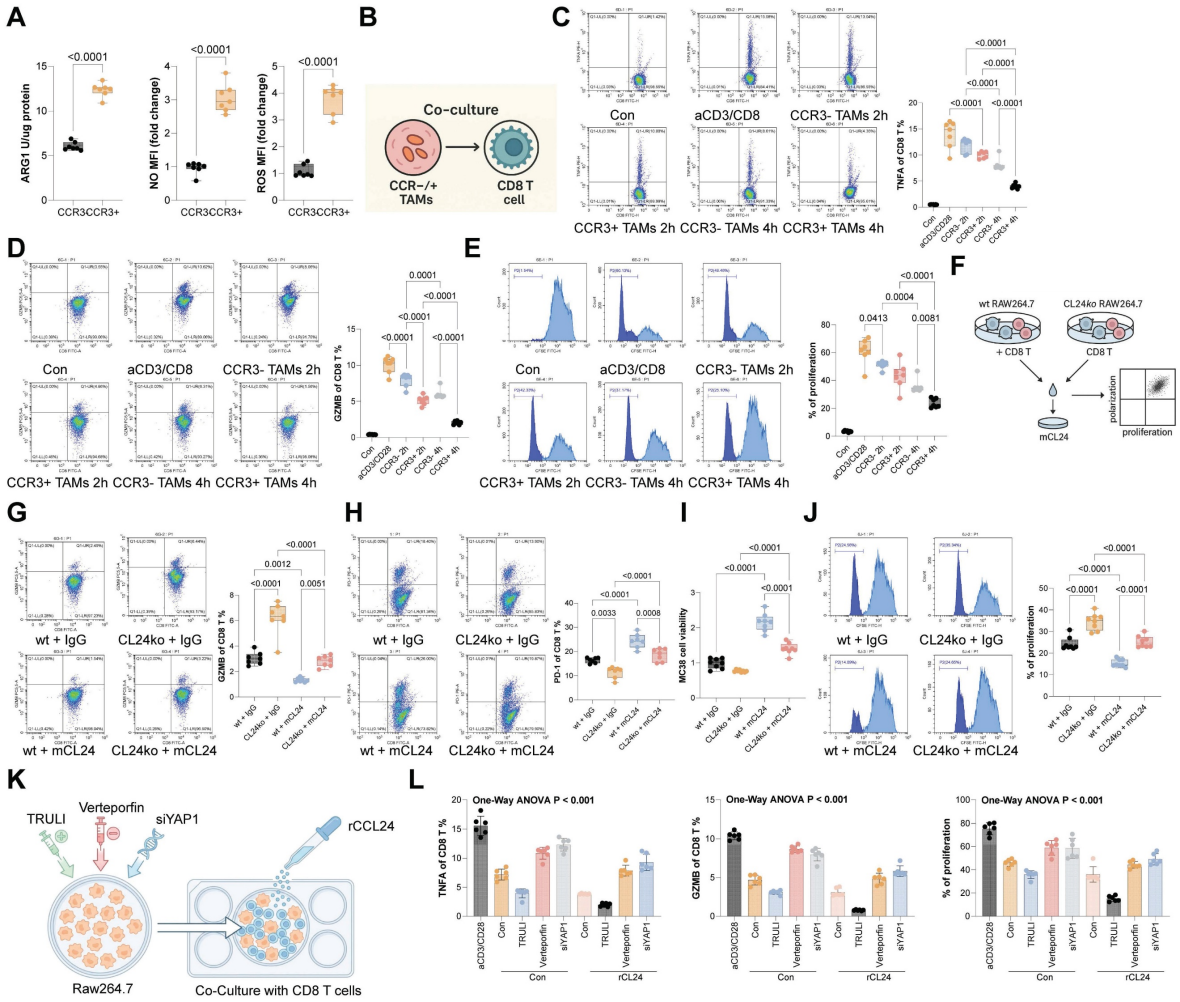
CCR3^+^ TAMs show stronger suppressive effects on CD8+ T cells over CCR3⁻ TAMs. A, Concentrations of ARG1, NO, and ROS secreted by CCR3⁻ or CCR3^+^ TAMs determined using ELISA kits. B, CCR3⁻ or CCR3^+^ TAMs were co-cultured with CD8⁺ T cells. C-D, Populations of GZMB^+^ (C) or TNFA^+^ (D) CD8^+^ T cells after co-culture determined using flow cytometry. E, Proliferation of CD8^+^ T cells after co-culture determined using CFSE labeling. F, WT or CCL24ko RAW264.7 cells were harvested and co-cultured with mouse CD8^+^ T cells. G-H, Populations of GZMB^+^ (G) or PD-1^+^ (H) CD8^+^ T cells after co-culture determined using flow cytometry. I, Analysis of CD8^+^ T cell cytotoxic effects on MC38 cells after co-culture with RAW264.7 cells. J, Proliferation of CD8^+^ T cells after co-culture determined using CFSE labeling. K, RAW264.7 macrophages were treated with TRULI (YAP1 activator), Verteporfin (YAP1 inhibitor), or siYAP1, and then co-cultured with activated CD8^+^ T cells in the presence/absence of mCCL24. (L) Quantification of GZMB+ and TNFA+ CD8+ T cells by flow cytometry. Cell experiments repeated 6-8 times. Data are presented as bars and dots. *p* < 0.05 was considered statistically significant.

Compared to CCR3⁻ TAMs, the CCR3^+^ ones exhibited substantially higher secretion of ARG1, NO, and ROS (Fig. 6A). To assess the impact of these TAMs on CD8^+^ T cell function, CCR3⁻ or CCR3^+^ TAMs were co-cultured with CD8⁺ T cells (Fig. 6B). Notably, CCR3^+^ TAMs exerted a significantly stronger suppressive effect on CD8^+^ T cell function compared to CCR3⁻ TAMs (Fig. 6C-D) and restricted CD8 T cell proliferation (Fig. 6E). To further validate, WT or CCL24^ko^ RAW264.7 cells were harvested and co-cultured with mouse CD8^+^ T cells, followed by the supplementation of mCCL24 (Fig. 6F). Notably, co-culturing with the CCL24^ko^ RAW264.7 cells substantially increased the population of effector CD8^+^ T cells (GZMB^+^) while reducing the population of exhausted CD8^+^ T cells (PD-1) (Fig. 6G-H). The cytotoxicity of these CD8^+^ T cells, after co-cultured with CCL24^ko^ RAW264.7 cells, was enhanced (Fig. 6I), accompanied by increased CD8^+^ T cell proliferation (Fig. 6J). However, these trends were largely counteracted by the supplementation of mCCL24 to the culture system (Fig. 6G-J). To definitively link YAP1 activation in TAMs to T cell suppression, we performed functional rescue experiments. Macrophages treated with TRULI (inducing constitutive YAP1 activation) exerted potent suppressive effects on CD8^+^ T cells, significantly reducing GZMB and TNFA expression even in the absence of CCL24 stimulation. Conversely, when YAP1 was silenced in macrophages using siYAP1 or inhibited pharmacologically with Verteporfin, the immunosuppressive effect of CCL24 was abrogated. These YAP1-deficient macrophages failed to suppress CD8^+^ T cells even when stimulated with recombinant CCL24 (Fig. 6K-L). These data confirm that YAP1 nuclear accumulation is the critical functional mediator enabling CCL24-stimulated TAMs to suppress anti-tumor immunity.

### Gracillin targets CCL24 and enhances antitumor immunity

**Figure 7:**
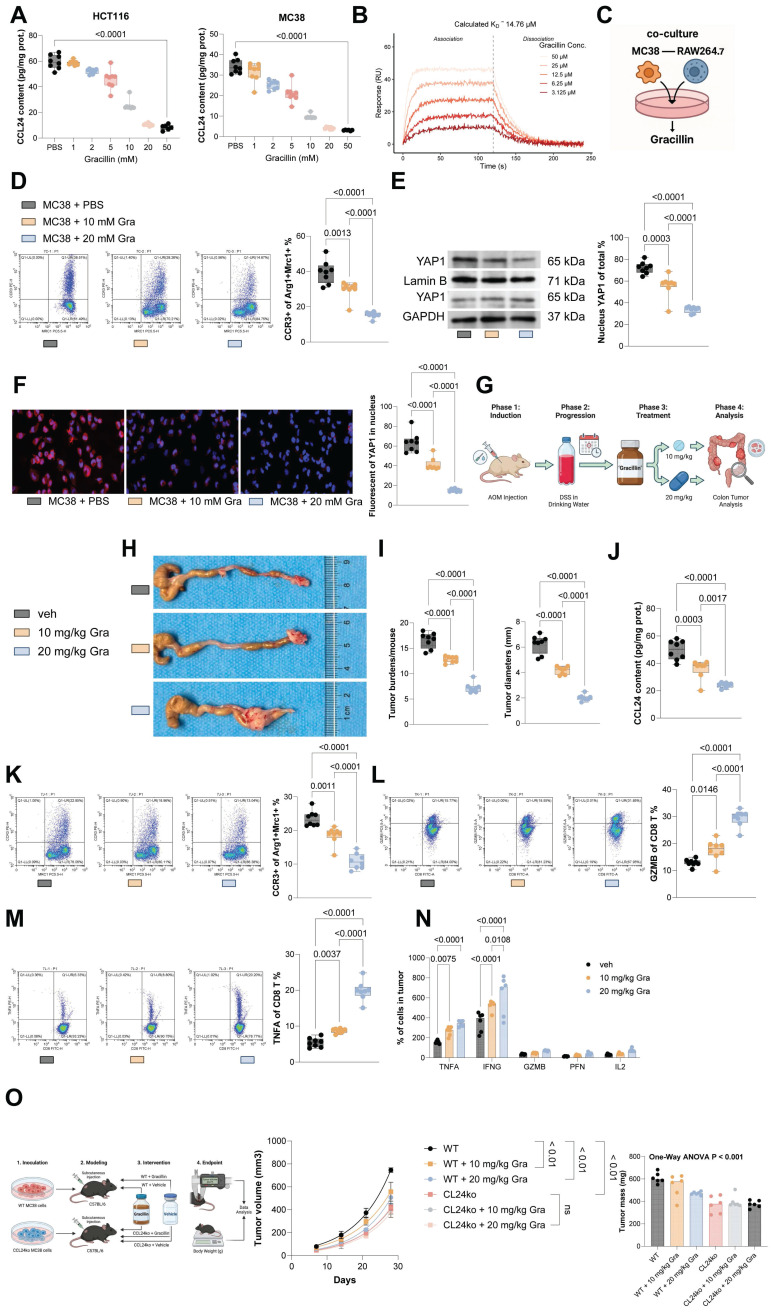
Gracillin targets CCL24 and enhances antitumor immunity. A, MC38 or HCT116 cells were treated with graded concentrations of Gracillin, followed by ELISA detection of CCL24 secretion. B, Representative sensorgrams showing the binding kinetics of Gracillin (at indicated concentrations) to immobilized recombinant CCL24 protein. The equilibrium dissociation constant (K_D_) indicates direct binding affinity. C, Gracillin was added to the co-culture system of MC38 cells and RAW264.7 cells at 10 mM or 20 mM. D, Number of CCR3^+^Arg1^+^Mrc1^+^ RAW264.7 cells after Gracillin addition determined using flow cytometry. E, Nuclear-cytoplasmic component separation and WB analysis to detect YAP1 sub-cellular localization in RAW264.7 cells after Gracillin addition, with Lamin B as the nuclear marker and GAPDH as the cytoplasmic marker. F, Immunofluorescence detection of YAP1 nuclear accumulation in RAW264.7 cells after Gracillin addition (Red fluorescence indicates YAP1 protein; Blue fluorescence indicates DAPI nuclear staining). G, Gracillin was then applied in the AOM/DSS-induced CRC mouse models at 10 or 20 mg/kg. H, Gross images of mouse colons. I, Tumor number and diameter in mouse colons. J, ELISA detection of CCL24 levels in mouse serum. K, Number of CCR3^+^Arg1^+^Mrc1^+^ numbers in mouse colon tissue determined using flow cytometry. L-M, Number of TNFA^+^ and GZMB^+^CD8^+^ T cell numbers in tumor tissue determined using flow cytometry. N, ELISA detection of TNFA, IFNG, GZMB, PFN, and IL2 levels in mouse serum. O, C57BL/6 mice were inoculated subcutaneously with WT or CCL24^ko^ MC38 cells and treated with Gracillin (20 mg/kg) or vehicle. Tumor volume and weight were recorded. Cell experiments repeated 6-8 times. Animal experiments include 6-8 mice per group. Data are presented as bars and dots. *p* < 0.05 was considered statistically significant.

To identify potential pharmacological inhibitors of CCL24, molecular docking was conducted, revealing Gracillin as a potential compound to target CCL24 (Fig. S6). Notably, treatment of MC38 or HCT116 cells with increasing concentrations of Gracillin significantly reduced CCL24 secretion (Fig. 7A). Subsequently, SPR assays were performed to confirm that this effect was mediated by direct physical interaction, we performed Surface Plasmon Resonance (SPR) assays. The results demonstrated that Gracillin binds directly to purified CCL24 protein in a dose-dependent manner, with a calculated binding affinity (K_D_) of approximately 19.25 μM (Fig. 7B). This confirms that Gracillin acts as a direct ligand for CCL24, likely compromising its stability or blocking its receptor-binding interface. In addition, Gracillin was added to the co-culture system of MC38 cells and RAW264.7 cells (Fig. 7C), which substantially reduced the population of immunosuppressive macrophages (Fig. 7D) and reduced nuclear accumulation of YAP1 (Fig. 7E-F). In addition, Gracillin was then applied in the AOM/DSS-induced CRC mouse models at 10 or 20 mg/kg (Fig. 7G). Notably, Gracillin treatment, particularly at a dose of 20 mg/kg, significantly reduced tumor incidence and size (Fig. 7H-I), lowered CCL24 levels in mouse serum (Fig. 7J), and reduced the number of CCR3^+^ TAMs in the tumor tissue (Fig. 7K). Conversely, CD8^+^ T cell numbers, as well as TNFA^+^ and GZMB^+^ CD8 T cells, were significantly increased (Fig. 7L-M). Serum levels of TNFA, IFNG, GZMB, PFN, and IL2 were also significantly elevated by the Gracillin treatment (Fig. 7N). To further verify that the anti-tumor efficacy of Gracillin is specifically mediated through CCL24 inhibition, rescue experiments were further performed using CCL24^ko^ MC38 tumors in C57BL/6 mice. As expected, Gracillin treatment significantly suppressed the growth of WT MC38 tumors. However, in mice bearing CCL24^ko^ tumors, where the therapeutic target is absent, Gracillin treatment failed to confer any additional inhibition of tumor growth compared to the vehicle-treated CCL24^ko^ group (Fig. 7O). This specific loss of efficacy in the knockout model strongly indicates that Gracillin exerts its anti-CRC effects primarily by blocking the CCL24 signaling axis rather than through off-target mechanisms. Importantly, we also evaluated the safety profile of Gracillin treatment *in vivo*. Continuous monitoring revealed no significant loss of body weight in Gracillin-treated mice compared to controls (Fig. S7A). Furthermore, histological examination of major organs, including the liver, kidney, heart, and lung, showed no observable pathological changes or toxicity at the therapeutic dose of 20 mg/kg (Fig. S7B). These findings suggest that Gracillin is well-tolerated and exhibits a favorable safety profile.

### Gracillin enhances the therapeutic efficacy of anti-cancer treatments

**Figure 8:**
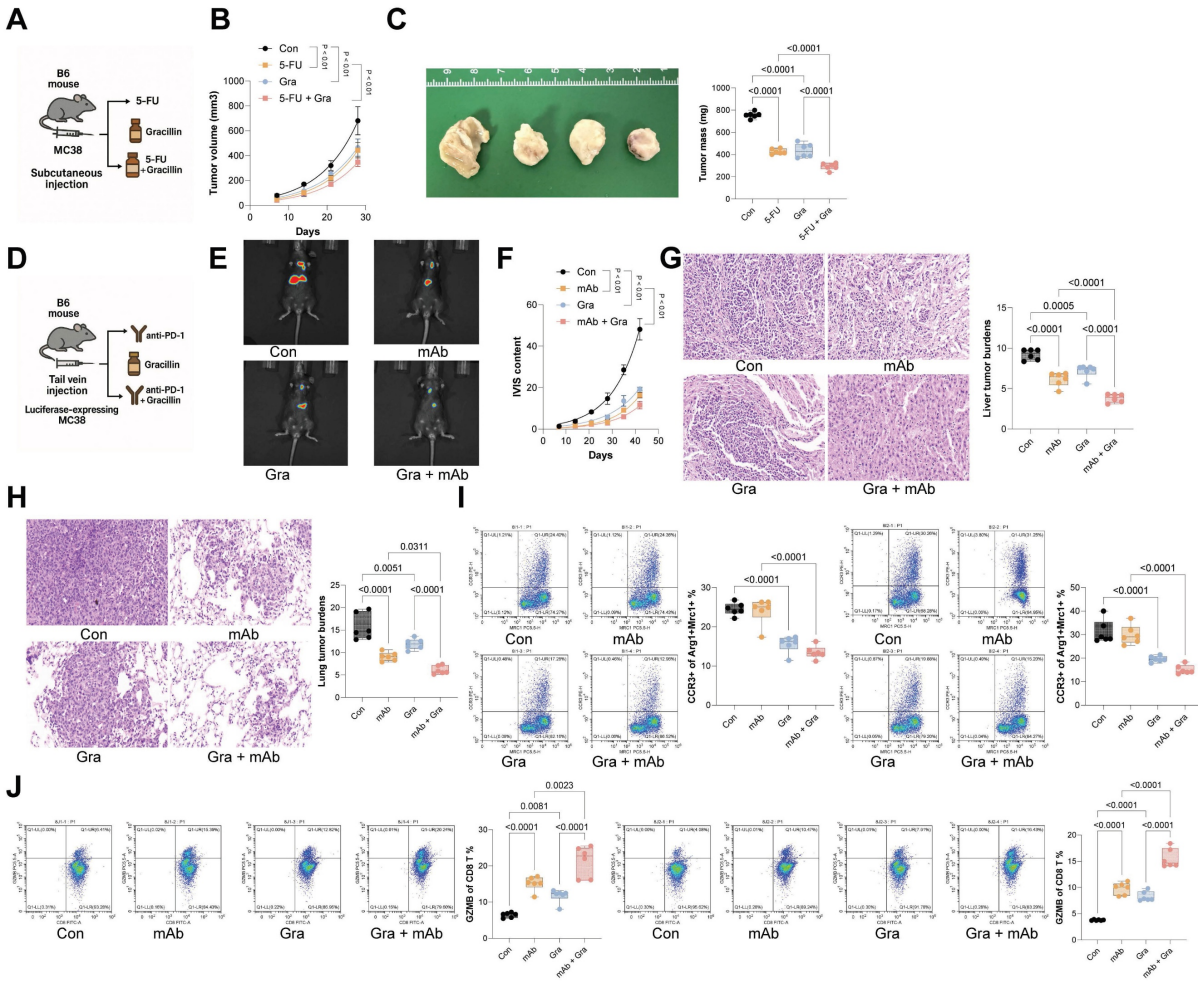
Gracillin enhances the therapeutic efficacy of anti-cancer treatments. A, Subcutaneous inoculation of MC38 cells into C57BL/6 mice, followed by treatment with 5-fluorouracil (5-FU) or Gracillin, or the combination of the two. B-C, Growth rate and weight of MC38 subcutaneous tumors. D, Tail vein inoculation of luciferase-expressing MC38 cells into C57BL/6 mice, followed by treatment with PD-1 mAb, Gracillin, or the combination of the two. E-F, Tumor cell dissemination and distribution in mice determined using the IVIS. G-H, Number of metastatic nodules formed by MC38 cells in mouse liver and lung tissues determined using HE staining. I-J, Numbers of CCR3^+^Arg1^+^Mrc1^+^ TAMs (I) or GZMB+CD8+ T cells (J) in lung and liver metastatic nodules determined using flow cytometry. Each group include 6-8 mice per group. Data are presented as bars and dots. *p* < 0.05 was considered statistically significant.

To further evaluate Gracillin's therapeutic effect in CRC, MC38 cells were inoculated subcutaneously into C57BL/6 mice, followed by treatment with 5-FU or Gracillin, or combination of the two (Fig. 8A). The sole treatment of either 5-FU or Gracillin substantially reduced the tumorigenic activity of MC38 cells in mice, with the combination strategy showing the most pronounced effect (Fig. 8B-C). Furthermore, considering Gracillin's effects on CCL24 inhibition and immune reactivation, we had luciferase-expressing MC38 cells inoculated via tail vein into C57BL/6 mice, followed by treatment with PD-1 mAb, Gracillin, or the two in combination (Fig. 8D). Gracillin showed significant synergistic effects with PD-1 mAb to reduce MC38 cell activity and dissemination *in vivo* (Fig. 8E-F). Additionally, the combination of Gracillin and PD-1 mAb significantly reduced the number of metastatic nodules formed by MC38 cells in mouse liver and lung tissues (Fig. 8G-H). Moreover, treatment of either PD-1 mAb or Gracillin significantly reduced the population of CCR3^+^ TAMs in the metastatic nodules (Fig. 8I) while increasing the population of GZMB^+^CD8^+^ T cells (Fig. 8J), with the combination treatment of these two showed the greatest efficacy.

## Discussion

Chemokines and their receptors, expressed by both tumor and stromal cells, have emerged as promising targets for immunotherapy in cancer treatment 33. This study reveals that the interaction between CCL24 and its receptor CCR3 significantly influences the recruitment of TAMs to the CRC TME, resulting in diminished CD8+ T cell cytotoxicity. Furthermore, we identified Gracillin, a novel compound, as a potential inhibitor of CCL24, capable of reprogramming the immunosuppressive TME to enhance anti-tumor immunity.

Bioinformatics analysis of the TCGA-COAD dataset, combined with IHC on CRC TMAs, demonstrated elevated CCL24 expression in CRC tissues. This finding aligns partially with prior studies indicating CCL24 upregulation in primary CRC tumor regions 27. The review by Lim also suggested that CCL24 is expressed in CRC cells, and CRC patients exhibit higher plasma levels of CCL24 34. Increased CCL24 levels have also been demonstrated in human hepatocellular carcinoma tissues 35, 36. However, the precise function of CCL24 in tumor immunity remains poorly understood. Our data from bioinformatics and TMA analyses also showed a strong association between elevated CCL24 levels, increased TAM infiltration, compromised T cell function, and worse prognosis in CRC patients. Supporting this, Olsen* et al.* reported that high CCL24 levels are specifically linked to CRC-related mortality 37. To explore this further, we developed primary CRC mouse models. Notably, both antibody-based CCL24 inhibition and colon epithelium-specific CCL24 conditional knockout (CCL24^cko^) in mice reduced AOM/DSS-induced colorectal tumorigenesis. The specific deletion of CCL24 in the tumor epithelium alone was sufficient to drastically reduce tumor burden. Although CCL24 can be expressed by various stromal components, including fibroblasts and endothelial cells, the profound reduction in tumorigenesis observed in these mice demonstrates that tumor-derived CCL24 is the dominant driver of the immunosuppressive phenotype in this context. In the study by Jin *et al.*, CCL24 overexpression promoted proliferation and mobility of hepatocellular carcinoma cells *in vitro* and augmented the tumorigenic activity of these cells in nude mice 36. However, in our study, CCL24^ko^ HCT116 and MC38 cells did not show significant changes in* in vitro* proliferation or invasion, and the tumorigenic potential of CCL24^ko^ MC38 cells was reduced in immunocompetent but not in immunodeficient nude mice. This discrepancy suggests that the function of CCL24 in tumors can be context-dependent. With the additional evidence that CD8^+^ T cell supplementation reduced the tumorigenic ability of CCL24^ko^ in nude mice, it can be inferred that the CCL24's tumor-promoting effects rely on immune regulation.

Interestingly, CCL24 knockout in MC38 cells did not directly alter the population of activated TNFA^+^ and GZMB^+^ CD8^+^ T cells. This negative finding is mechanistically significant, as it effectively rules out a direct receptor-ligand interaction between CCL24 and T cells in this context. CCL24 primarily signals through CCR3, a receptor primarily expressed on the surface of eosinophils, basophils, and Th2 cells 38. However, the scRNA-seq analysis of CCL24^cko^ mice revealed a reduced macrophage population and increased CD8^+^ T cell numbers, with no significant changes in eosinophil counts. In fact, several pieces of evidence have demonstrated CCR3-dependent recruitment of TAMs to the tumor niche 39. This is also true for CRC, where CCL26 upregulation promotes TAM infiltration via CCR3 binding 40. CellChat analysis further supported that CRC cells communicate with macrophages through the CCL24-CCR3 axis. We propose that CCL24, secreted by CRC cells, binds to CCR3 on macrophages, driving TAM polarization and reshaping the TME. Indeed, CCL24 knockout in human and mouse CRC cells reduced immunosuppressive TAM populations in co-culture experiments. Additionally, reduced CCR3 staining was observed in tumor tissues from both ACl24-treated and CCL24^cko^ mice, verifying the recruitment of CCR3^+^ TAMs by CCL24^+^ tumors during CRC development.

Immunosuppressive TAMs are known to impair CD8^+^ T cell cytotoxicity by expressing surface proteins or secreting factors such as IL-10, TGF-β, and PD-L1, which undermine immune surveillance 41. These TAMs also recruit other immunosuppressive cells, such as regulatory T cells, further inhibiting T cell activity 42, 43. Thus, CCL24 likely promotes CD8^+^ T cell exhaustion in the CRC TME indirectly by recruiting CCR3^+^ TAMs. In co-culture experiments, CCR3^+^ TAMs exhibited stronger suppression of CD8^+^ T cell activity compared to CCR3^-^ TAMs and expressed higher levels of immunosuppressive markers like ARG1, NO, and ROS. Therefore, CCR3⁺ macrophages might represent a more M2-like phenotype. CCR3 is known to promote Th2 cytokine secretion 44, which enhances TAM immunosuppression 45. Prior studies have linked CCR3 expression to M2 macrophage polarization 46, 47. Our RNA-seq analysis and GSEA revealed that DEGs in CCL24^cko^ TAMs were enriched in the Hippo signaling pathway, a conserved pathway regulating cell fate, organ size, tissue regeneration, and tumorigenesis 48, 49. Inhibition of Hippo signaling leads to nuclear translocation of YAP1 and its paralog TAZ, promoting tumor cell proliferation and therapy resistance 50. YAP1 overexpression is oncogenic in multiple tissues, including the colon 51, and influences immune cell activity, including macrophages and regulatory T cells 52, 53. YAP1 also promotes M2 macrophage polarization 54 and inhibits CD8^+^ T cell infiltration 55 in solid tumors. In our study, co-culture with CRC cells increased YAP1 nuclear translocation in macrophages, while CCL24^cko^ TAMs showed reduced YAP1 enrichment near the transcription start sites of genes like Arg1, Mrc1, Chi3l3, and Retnla, indicating that YAP1 activation contributes to CCL24-CCR3-mediated immunosuppression in CRC. It should also be noted that TAMs exhibit significant ontogenic heterogeneity, originating either from tissue-resident progenitors or recruited peripheral blood monocytes. The observation that CCL24 specifically induces the migration of monocytes *in vitro*, combined with the significant reduction in TAM burden following CCL24 blockade *in vivo*, supports a mechanism of active recruitment from the circulation. Furthermore, our data establish CCR3 as a marker for a distinct, functionally specialized macrophage subset. Transcriptomic and functional comparisons revealed that, unlike the heterogeneous CCR3⁻ population, CCR3^+^ TAMs possess a unique molecular signature enriched in Hippo signaling and exhibit a hyper-suppressive phenotype characterized by elevated ROS, NO, and Arg1 production. Thus, CCR3 expression delineates a specific subpopulation of TAMs that are central to maintaining the immunosuppressive niche in CRC.

Targeting the CCL24-CCR3 interaction offers a promising approach to reverse immunosuppression in the CRC TME. The molecular docking analysis revealed Gracillin as a molecule showing high affinity with CCL24, and the SPR assays confirmed a direct, dose-dependent binding of Gracillin to CCL24. Gracillin is a natural steroidal saponin component abundant in several Dioscorea species 28. It has shown potential in suppressing proliferation and metastasis in various cancers 28, 56-58 and activates the Hippo pathway to reduce YAP1 levels in tumors 59. In CRC, Gracillin has shown potent effect on suppressing viability and migration while promoting apoptosis in cell lines like HCT116, RKO, and SW480 60. Its role in immune modulation, however, remains unexplored. Our findings demonstrate that Gracillin treatment dose-dependently reduced CCL24 secretion by MC38 and HCT116 cells, decreasing immunosuppressive marker expression in co-cultured TAMs. *In vivo*, Gracillin treatment lowered CCL24 levels in AOM/DSS-treated mice, reducing CCR3^+^ TAM populations and restoring activated CD8^+^ T cell numbers. However, the mechanism underpinning the inhibitory effect of Gracillin on CCL24 secretion remained elusive. We propose that Gracillin binding to CCL24 may induce conformational changes that may destabilize the protein, leading to its intracellular degradation or enhanced clearance. Additionally, it is possible that Gracillin binds to the same epitope recognized by the detection antibody, resulting in reduced detection levels, which would align with its ability to block protein-protein interactions. However, the precise mechanisms await further investigation. Additionally, Gracillin showed synergistic effects with 5-FU and anti-PD-1 therapies in mice with allograft tumors, without causing significant systemic toxicity or cachexia, highlighting its potential for CRC treatment.

In summary, this study demonstrates that CCL24, overexpressed in CRC cells, recruits CCR3^+^ TAMs to the TME, suppressing CD8^+^ T cell activity and cytotoxicity. Gracillin, an anti-cancer compound, counteracts this immunosuppressive cascade, offering a promising strategy to enhance immune responses in CRC therapy.

## Supplementary Material

Supplementary figures.
