# Supplementary material for: CCL24 recruits CCR3+ TAMs to promote immunosuppression via YAP1 activation and serves as a therapeutic target for Gracillin in colorectal cancer
Source: Int J Biol Sci. 2026 Jan 30;22(4):2157–76. doi: 10.7150/ijbs.127341 (PMC12905639; doi:10.7150/ijbs.127341)
Supplement: Supplementary file 1 — Supplementary figures. [file ijbsv22p2157s1.pdf]

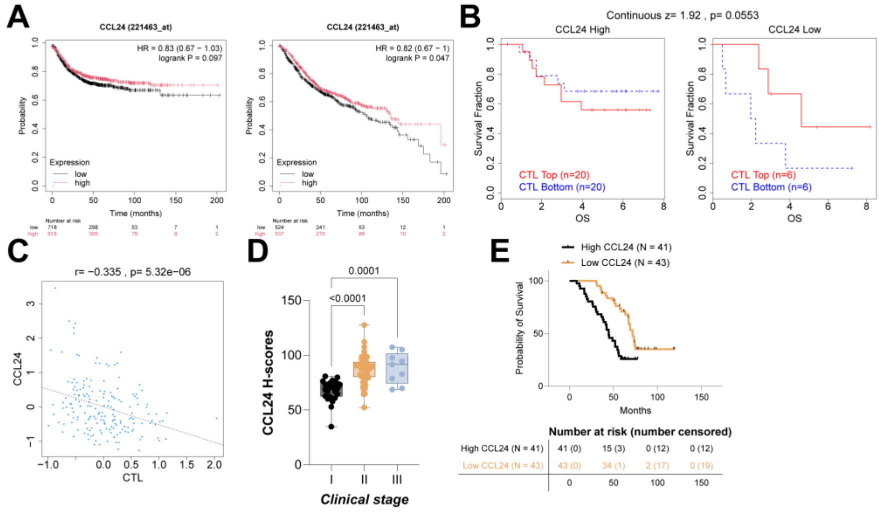

Fig S1. CCL24 is linked to unfavorable prognosis in CRC patients. A, Kaplan-Meier analysis of the correlation between CCL24 expression and overall survival and progression-free survival in the TCGA-COAD database. B-C, Correlation analysis of CCL24 with T cell dysfunction and CTL infiltration in the TIDE dataset. D-E, Analysis of CCL24 staining intensity correlation with clinical staging and overall survival in 84 CRC TMAs. TMA includes 84 pairs of tissue samples. Data are presented as bars and dots. $p < 0.05$ was considered statistically significant.

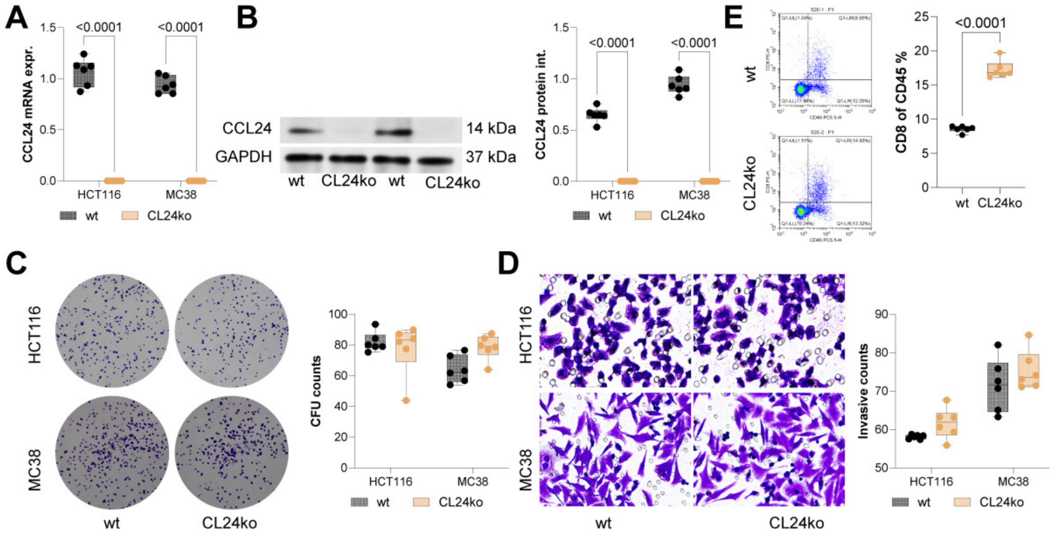

Fig S2. CCL24 knockout does not affect growth of CRC cells *in vitro*. CCL24^{ko} HCT116 and MC38 cell lines were generated using CRISPR/Cas9 technique. A-B, qPCR and WB analyses to detect CCL24 mRNA and protein levels in WT and CCL24^{ko} HCT116 and MC38 cells. C, Colony formation assays to analyze cell proliferation of HCT116 and MC38 cells. D, Transwell assays to analyze

invasion of HCT116 and MC38 cells. E, Number of CD8^{+} T cells in tumors formed by CCL24^{ko} MC38 cells in C57BL/6 mice determined using flow cytometry. Cell experiments repeated 6-8 times. Animal experiments include 6-8 mice per group. Data are presented as bars and dots. $p < 0.05$ was considered statistically significant.

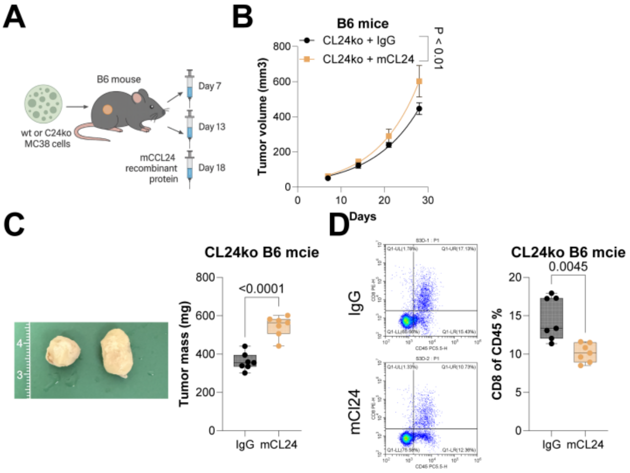

Fig S3. Mouse CCL24 recombinant protein restores tumorigenesis of CCL24^{ko} MC38 cells in mice. A, C57BL/6 mice implanted CCL24^{ko} MC38 cells were further treated with mCCL24. B-C, Analysis of MC38 cell growth rate and tumor weight in mice. D, Population of CD8^{+} T cells in mice determined using flow cytometry. Each group includes 6-8 mice. Data are presented as bars and dots. $p < 0.05$ was considered statistically significant.

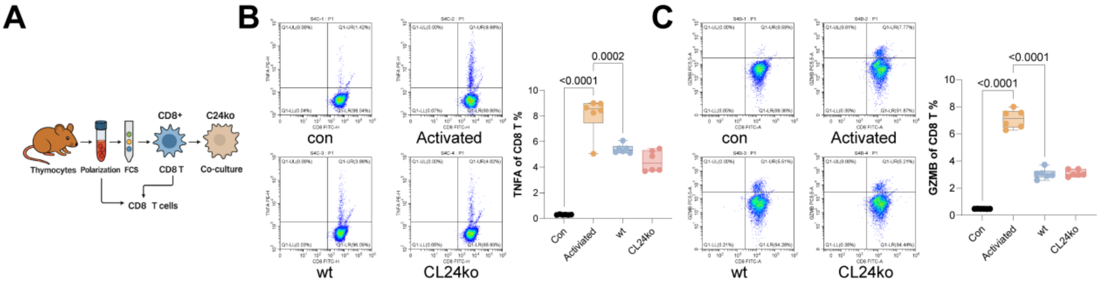

Fig S4. CCL24 expression in cancer cells does not directly affect T cell activity. A, CD8^{+} T cells were extracted from thymocytes or C57BL/6 mice, activated with CD3/CD28 antibodies, sorted by flow cytometry, and co-cultured with WT or CL24^{ko} MC38 cells *in vitro*. B-C, Populations of TNFA^{+} (B) and GZMB^{+} (C) CD8^{+} T cells after co-culture with cancer cells determined using flow cytometry.

Cell experiments repeated 6-8 times. Data are presented as bars and dots. $p < 0.05$ was considered statistically significant.

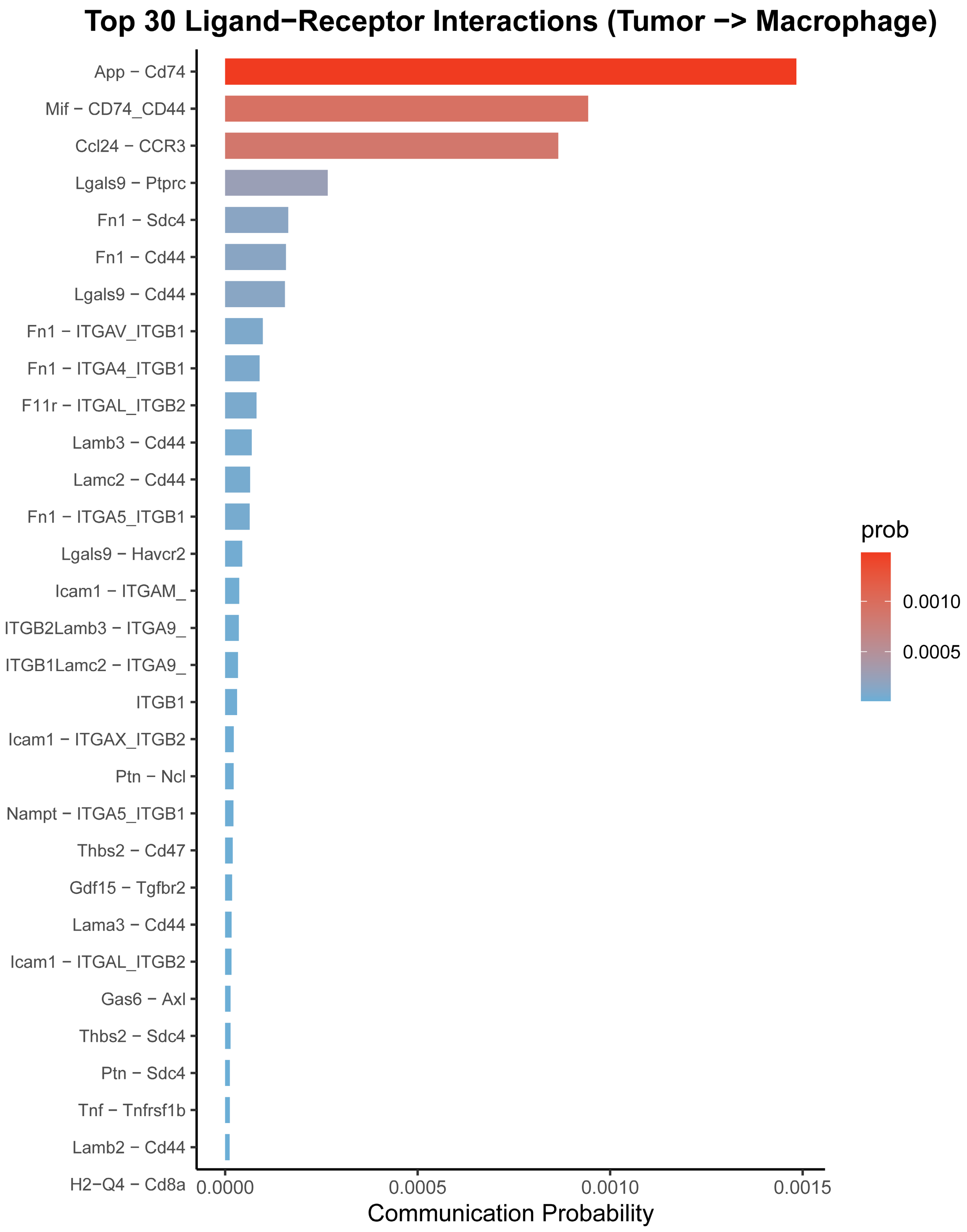

Fig S5. CellChat Analysis of the Top 30 signaling pathways between tumor cells and macrophages.

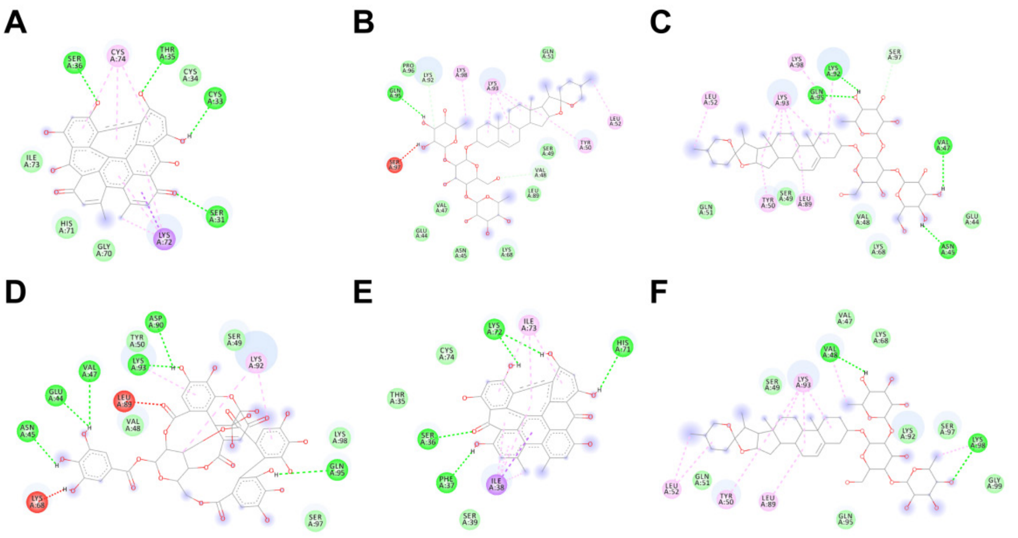

Fig S6. AutoDock Vina Analysis of Small Molecule Drugs Binding to CCL24. A, Hypericin (Binding Affinity, -8.3). B, Dioscin (Binding Affinity, -8.8). C, Gracillin (Binding Affinity, -8.9). D, Geraniin (Binding Affinity, -8.4). E, L-asparaginase (Binding Affinity, -8.2). F, CHEMBL2036082 (Binding Affinity, -8.5).

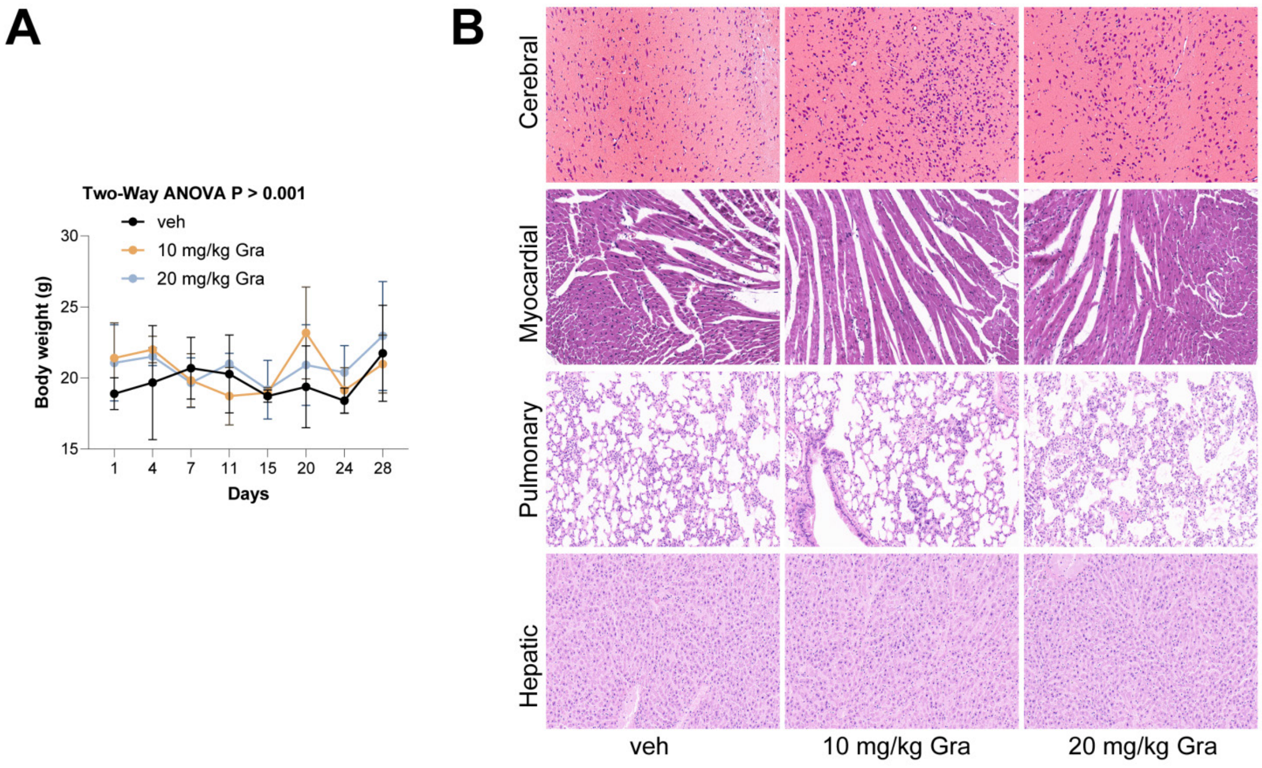

Fig S7. Safety evaluation of Gracillin in vivo. (A) Body weight changes of C57BL/6 mice during the course of Gracillin treatment. (B) Representative HE staining images of major organs (Heart, Liver, Lung, Kidney) harvested from vehicle- and Gracillin-treated mice at the endpoint. Scale bar = 100 μm. Note the absence of tissue damage or necrosis.

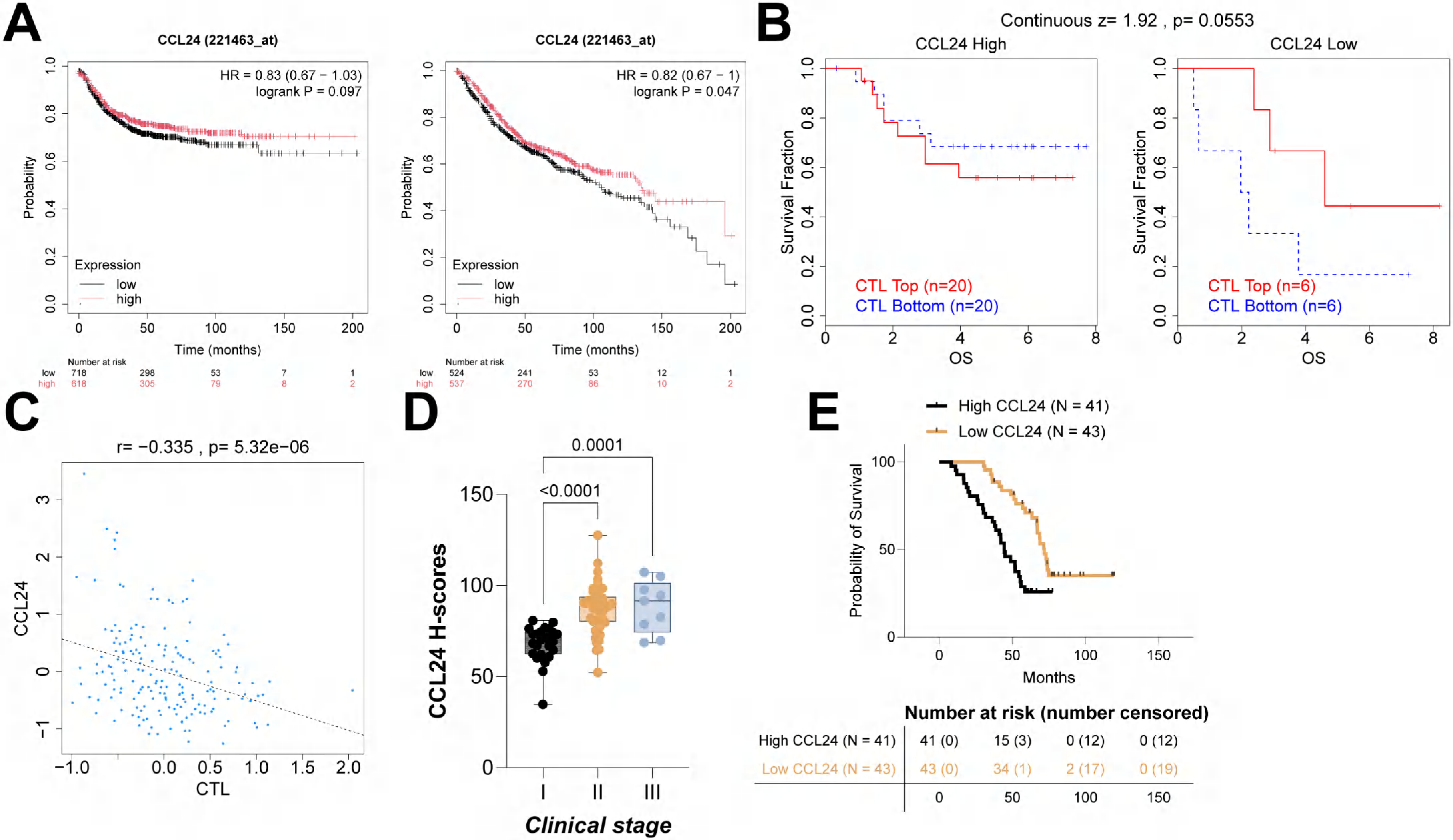
A
CCL24 (221463_at)
HR = 0.83 (0.67 − 1.03)
logrank P = 0.097
Probability
Time (months)
Expression
low
high
Number at risk
low 718 298 53 7 1
high 618 305 79 8 2
CCL24 (221463_at)
HR = 0.82 (0.67 − 1)
logrank P = 0.047
Number at risk
low 524 241 53 12 1
high 537 270 86 10 2
B
Continuous z= 1.92 , p= 0.0553
CCL24 High
CCL24 Low
Survival Fraction
OS
CTL Top (n=20)
CTL Bottom (n=20)
CTL Top (n=6)
CTL Bottom (n=6)
C
r= −0.335 , p= 5.32e−06
CCL24
CTL
D
0.0001
<0.0001
CCL24 H-scores
I
II
III
Clinical stage
E
High CCL24 (N = 41)
Low CCL24 (N = 43)
Probability of Survival
Months
Number at risk (number censored)
High CCL24 (N = 41) 41 (0) 15 (3) 0 (12) 0 (12)
Low CCL24 (N = 43) 43 (0) 34 (1) 2 (17) 0 (19)
0 50 100 150

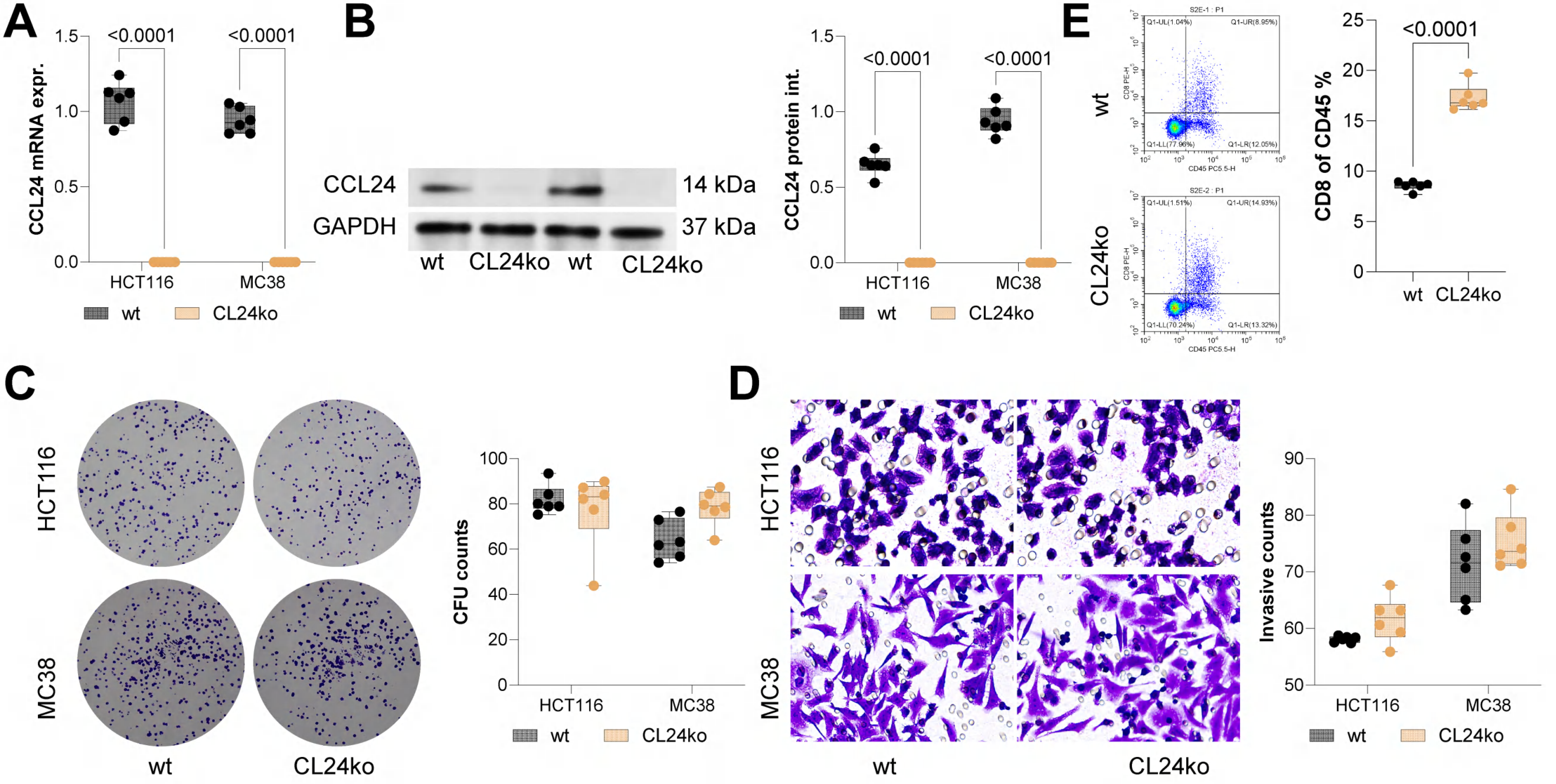

A
B
C
D
E
CCL24 mRNA expr.
<0.0001
<0.0001
HCT116
MC38
wt
CL24ko
CCL24
GAPDH
14 kDa
37 kDa
wt
CL24ko
wt
CL24ko
CCL24 protein int.
<0.0001
<0.0001
HCT116
MC38
wt
CL24ko
S2E-1 : P1
S2E-2 : P1
CD8 PE-H
CD45 PC5.5-H
Q1-UL(1.04%)
Q1-UR(8.95%)
Q1-LL(77.96%)
Q1-LR(12.05%)
Q1-UL(1.51%)
Q1-UR(14.93%)
Q1-LL(70.24%)
Q1-LR(13.32%)
CD8 of CD45 %
<0.0001
wt
CL24ko
HCT116
MC38
wt
CL24ko
CFU counts
HCT116
MC38
wt
CL24ko
HCT116
MC38
wt
CL24ko
Invasive counts
HCT116
MC38
wt
CL24ko

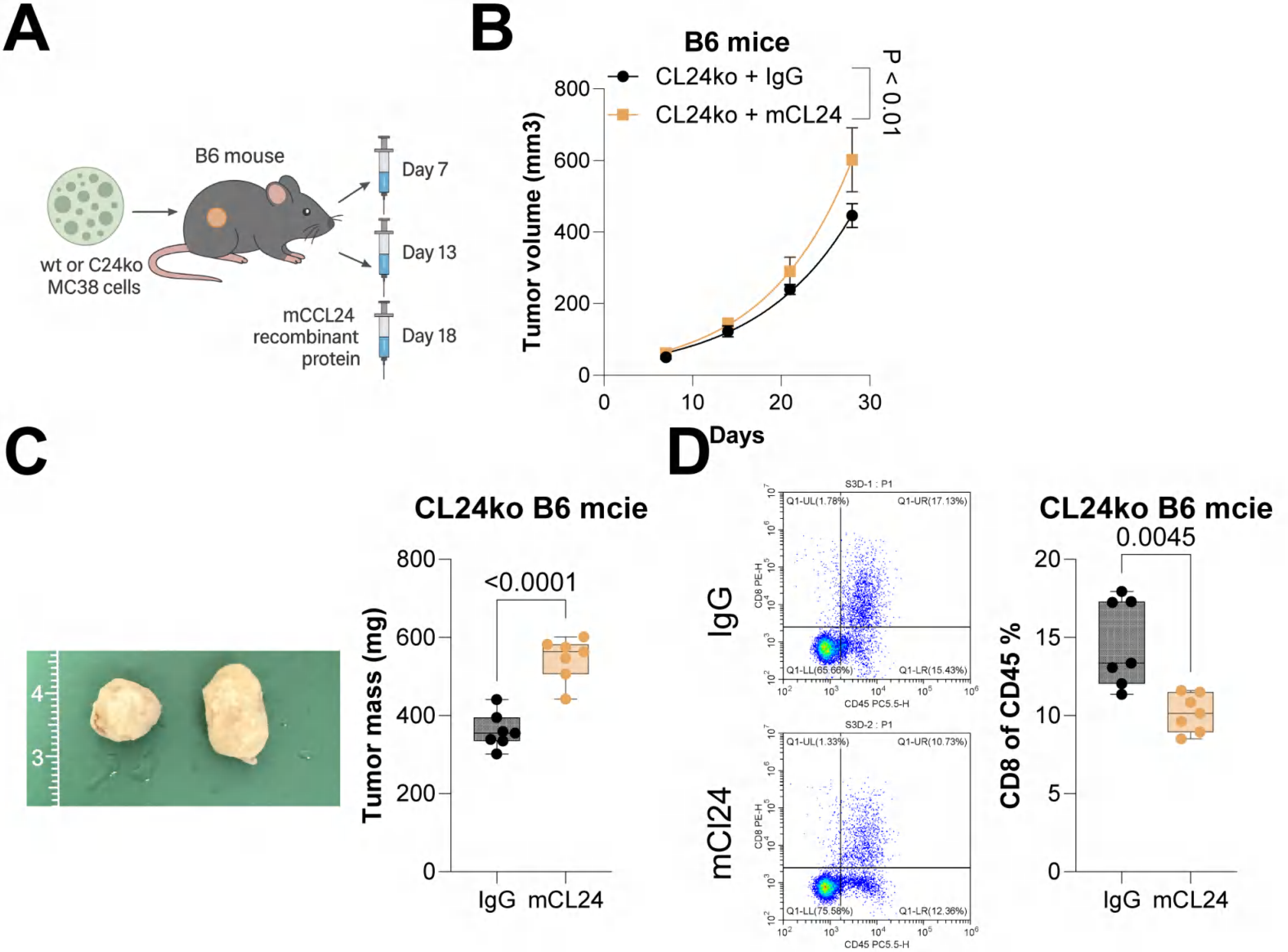
A
B6 mouse
wt or C24ko MC38 cells
Day 7
Day 13
Day 18
mCCL24 recombinant protein
B
B6 mice
CL24ko + IgG
CL24ko + mCL24
P < 0.01
Tumor volume (mm3)
800
600
400
200
0
0
10
20
30
Days
C
4
3
CL24ko B6 mcie
<0.0001
Tumor mass (mg)
800
600
400
200
0
IgG
mCL24
D
IgG
mCl24
S3D-1 : P1
Q1-UL(1.78%)
Q1-UR(17.13%)
Q1-LL(65.66%)
Q1-LR(15.43%)
S3D-2 : P1
Q1-UL(1.33%)
Q1-UR(10.73%)
Q1-LL(75.58%)
Q1-LR(12.36%)
CD8 PE-H
CD45 PC5.5-H
CL24ko B6 mcie
0.0045
CD8 of CD45 %
20
15
10
5
0
IgG
mCL24

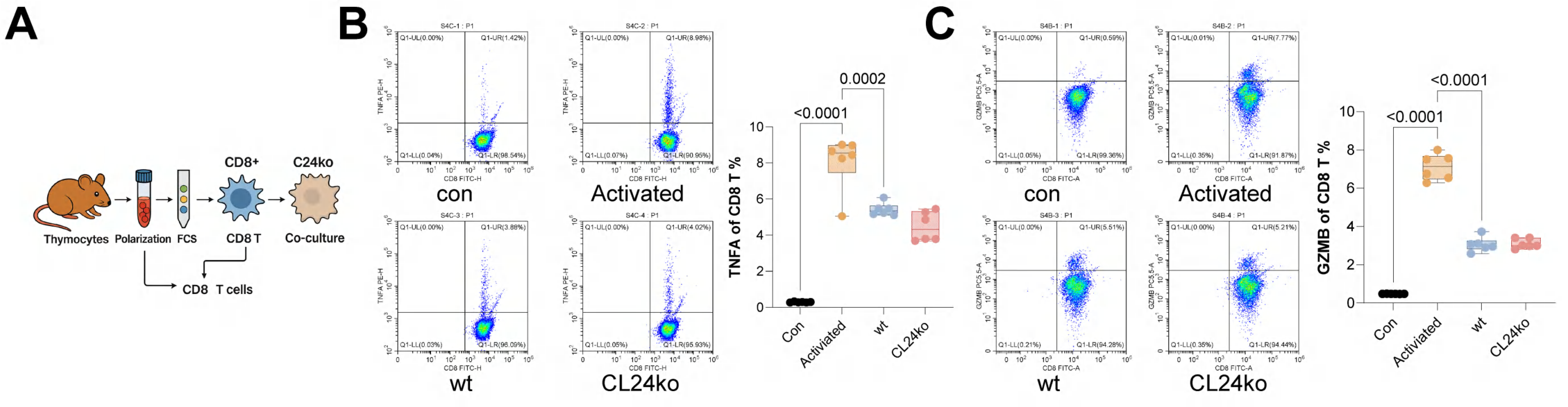
A
Thymocytes
Polarization
FCS
CD8+
CD8 T
C24ko
Co-culture
CD8 T cells
B
con
Activated
wt
CL24ko
TNFA of CD8 T %
<0.0001
0.0002
Con
Activated
wt
CL24ko
C
con
Activated
wt
CL24ko
GZMB of CD8 T %
<0.0001
<0.0001
Con
Activated
wt
CL24ko

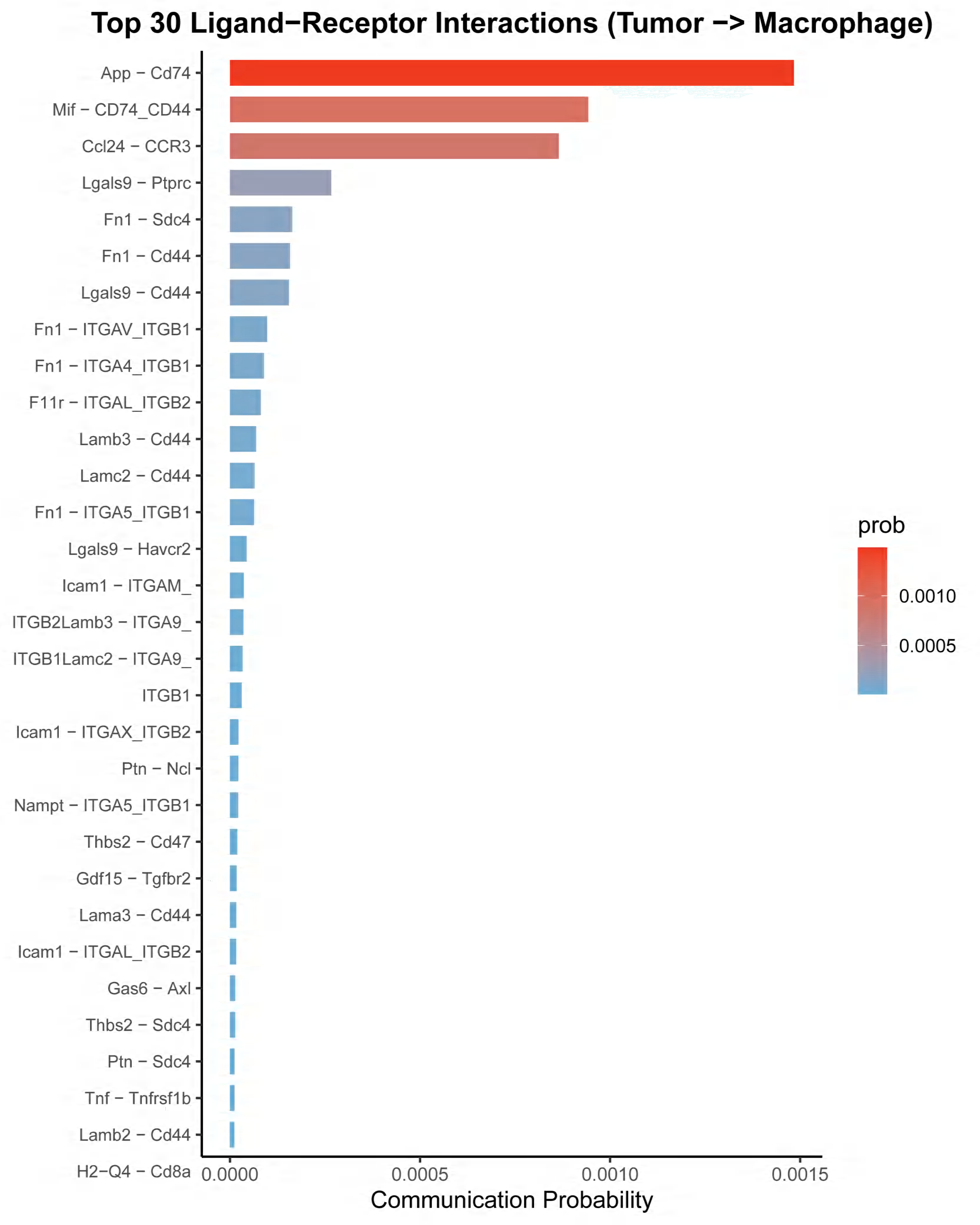
Top 30 Ligand−Receptor Interactions (Tumor −> Macrophage)
App − Cd74
Mif − CD74_CD44
Ccl24 − CCR3
Lgals9 − Ptprc
Fn1 − Sdc4
Fn1 − Cd44
Lgals9 − Cd44
Fn1 − ITGAV_ITGB1
Fn1 − ITGA4_ITGB1
F11r − ITGAL_ITGB2
Lamb3 − Cd44
Lamc2 − Cd44
Fn1 − ITGA5_ITGB1
Lgals9 − Havcr2
Icam1 − ITGAM_
ITGB2Lamb3 − ITGA9_
ITGB1Lamc2 − ITGA9_
ITGB1
Icam1 − ITGAX_ITGB2
Ptn − Ncl
Nampt − ITGA5_ITGB1
Thbs2 − Cd47
Gdf15 − Tgfbr2
Lama3 − Cd44
Icam1 − ITGAL_ITGB2
Gas6 − Axl
Thbs2 − Sdc4
Ptn − Sdc4
Tnf − Tnfrsf1b
Lamb2 − Cd44
H2−Q4 − Cd8a
0.0000
0.0005
0.0010
0.0015
Communication Probability
prob
0.0010
0.0005

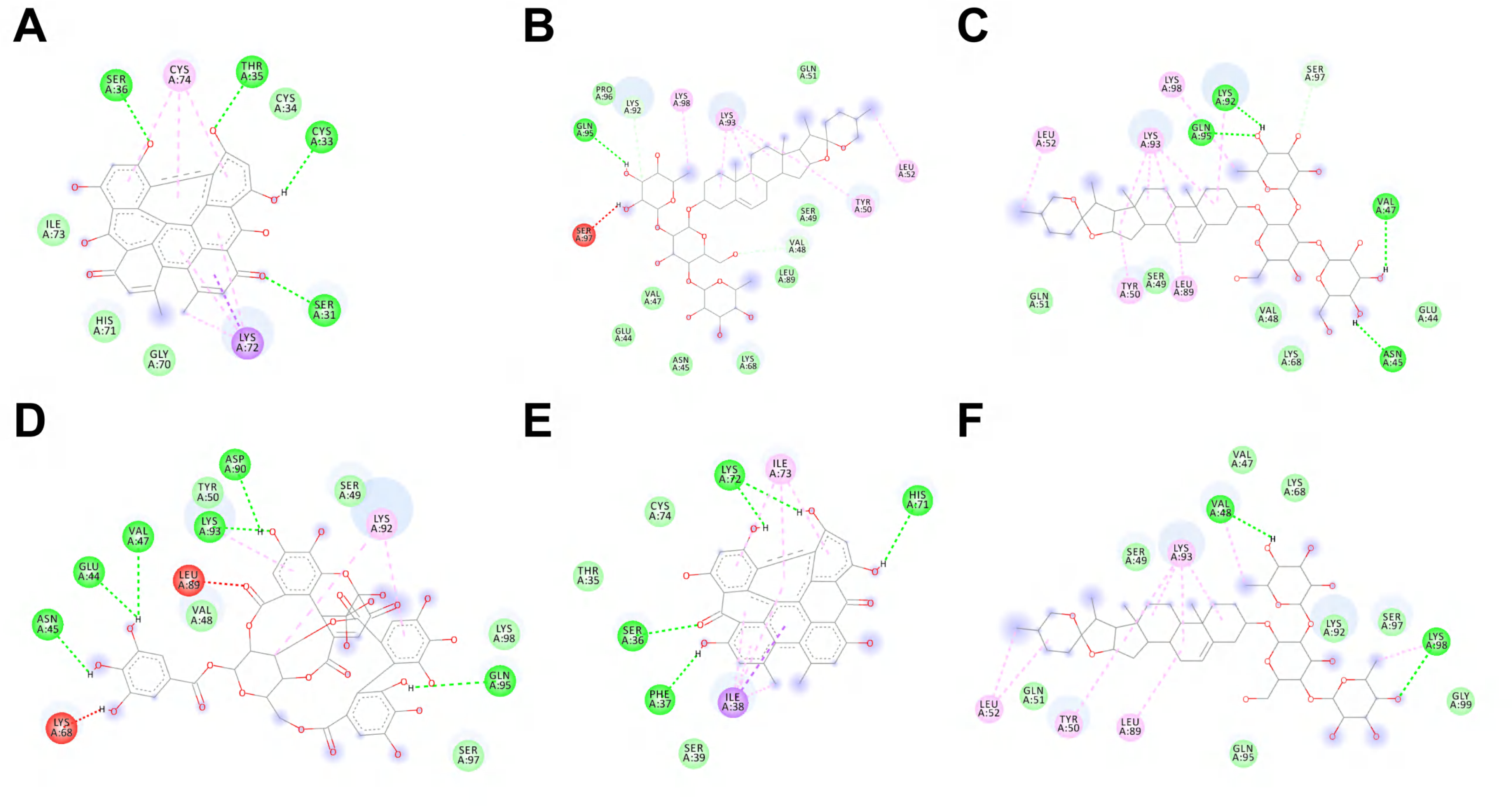
A
SER A:36
CYS A:74
THR A:35
CYS A:34
CYS A:33
ILE A:73
HIS A:71
GLY A:70
LYS A:72
SER A:31
B
PRO A:96
LYS A:92
LYS A:98
GLN A:51
GLN A:95
LYS A:93
LEU A:52
TYR A:50
SER A:49
SER A:97
VAL A:48
LEU A:89
VAL A:47
GLU A:44
ASN A:45
LYS A:68
C
LYS A:98
LYS A:92
SER A:97
LEU A:52
LYS A:93
GLN A:95
VAL A:47
TYR A:50
SER A:49
LEU A:89
GLN A:51
VAL A:48
GLU A:44
LYS A:68
ASN A:45
D
ASP A:90
TYR A:50
SER A:49
LYS A:93
LYS A:92
VAL A:47
GLU A:44
LEU A:89
VAL A:48
ASN A:45
LYS A:98
GLN A:95
LYS A:68
SER A:97
E
LYS A:72
ILE A:73
HIS A:71
CYS A:74
THR A:35
SER A:36
PHE A:37
ILE A:38
SER A:39
F
VAL A:47
LYS A:68
VAL A:48
SER A:49
LYS A:93
LYS A:92
SER A:97
LYS A:98
LEU A:52
GLN A:51
TYR A:50
LEU A:89
GLY A:99
GLN A:95

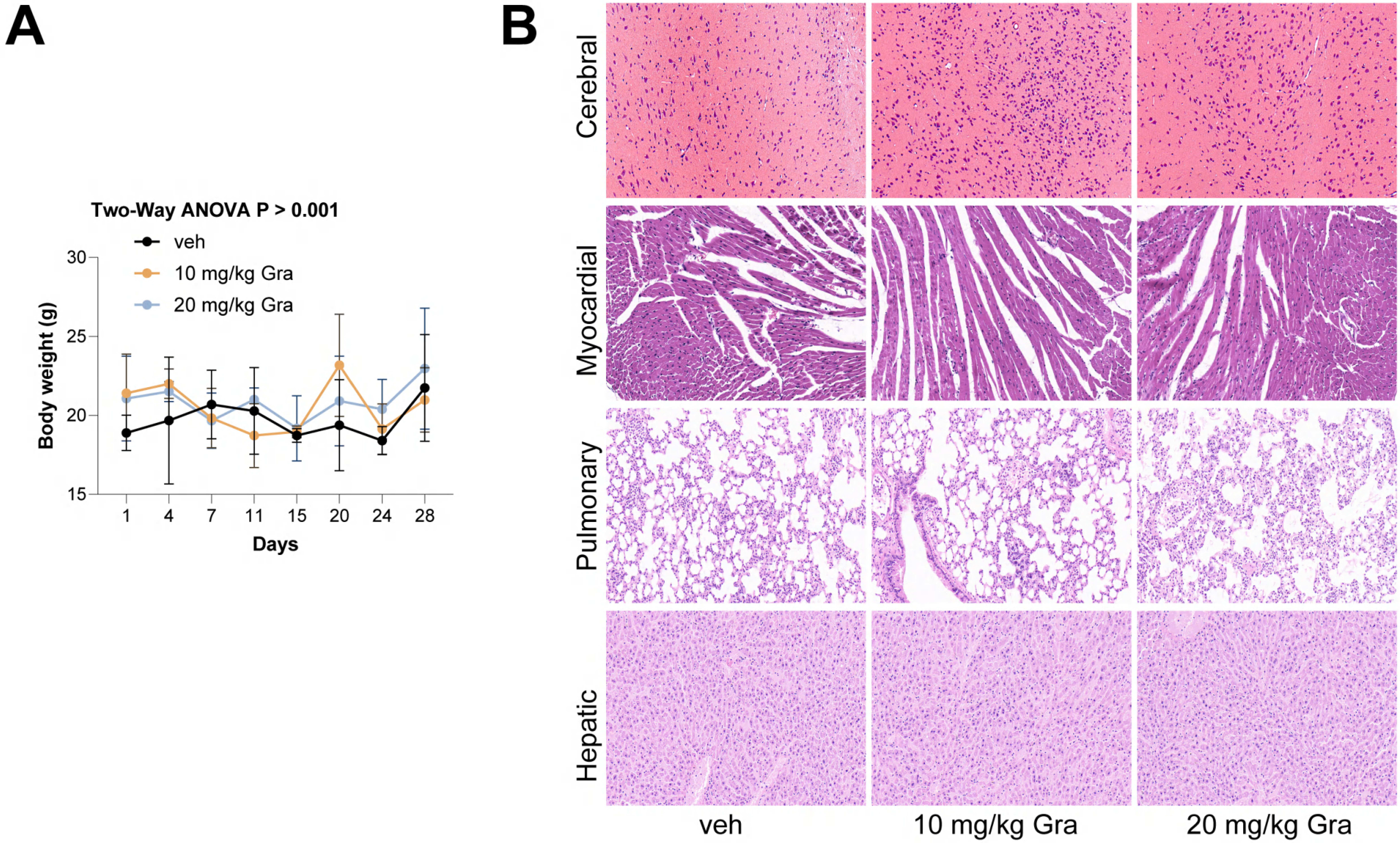
A
Two-Way ANOVA P > 0.001
veh
10 mg/kg Gra
20 mg/kg Gra
Body weight (g)
30
25
20
15
1
4
7
11
15
20
24
28
Days
B
Cerebral
Myocardial
Pulmonary
Hepatic
veh
10 mg/kg Gra
20 mg/kg Gra
